# Efficient Refinement of Complex Structures of Flexible Histone Peptides Using Post-Docking Molecular Dynamics Protocols

**DOI:** 10.3390/ijms25115945

**Published:** 2024-05-29

**Authors:** Bayartsetseg Bayarsaikhan, Balázs Zoltán Zsidó, Rita Börzsei, Csaba Hetényi

**Affiliations:** 1 Pharmacoinformatics Unit, Department of Pharmacology and Pharmacotherapy, Medical School, University of Pécs, Szigeti út 12, H-7624 Pécs, Hungary; bayartsetseg704@yahoo.com (B.B.); zsido.balazs@pte.hu (B.Z.Z.); rita.borzsei@aok.pte.hu (R.B.); 2 National Laboratory for Drug Research and Development, Magyar tudósok krt. 2, H-1117 Budapest, Hungary

**Keywords:** peptide, histones, docking, refinement, molecular dynamics, water

## Abstract

Histones are keys to many epigenetic events and their complexes have therapeutic and diagnostic importance. The determination of the structures of histone complexes is fundamental in the design of new drugs. Computational molecular docking is widely used for the prediction of target–ligand complexes. Large, linear peptides like the tail regions of histones are challenging ligands for docking due to their large conformational flexibility, extensive hydration, and weak interactions with the shallow binding pockets of their reader proteins. Thus, fast docking methods often fail to produce complex structures of such peptide ligands at a level appropriate for drug design. To address this challenge, and improve the structural quality of the docked complexes, post-docking refinement has been applied using various molecular dynamics (MD) approaches. However, a final consensus has not been reached on the desired MD refinement protocol. In this present study, MD refinement strategies were systematically explored on a set of problematic complexes of histone peptide ligands with relatively large errors in their docked geometries. Six protocols were compared that differ in their MD simulation parameters. In all cases, pre-MD hydration of the complex interface regions was applied to avoid the unwanted presence of empty cavities. The best-performing protocol achieved a median of 32% improvement over the docked structures in terms of the change in root mean squared deviations from the experimental references. The influence of structural factors and explicit hydration on the performance of post-docking MD refinements are also discussed to help with their implementation in future methods and applications.

## 1. Introduction

Fast molecular docking methods are widely used tools of drug design and molecular engineering [1]. The docking procedure aims to search the target–ligand conformational space at a reasonable computational cost to predict the most probable binding mode (position, orientation, and conformation) of ligands in the binding pocket of a target [2,3]. While docking is undoubtedly a leading technique, it still faces persistent challenges [4,5,6,7,8,9], especially when it comes to large, flexible peptide ligands. However, peptides mediate up to 40% of naturally occurring protein–protein interactions and play a central role in various cellular processes, including signal transduction, transcriptional regulation, immune response, and oncology [10,11,12,13,14]. Structural models of peptide–protein complexes have been used to design inhibitory peptides and peptidomimetics that modulate protein–protein interactions involved in various disease pathways [14,15,16,17,18,19,20]. In addition to their high specificity and relatively low toxicity [21,22,23,24], peptides have been able to successfully target protein complexes such as transcription factors, which were considered undruggable by small molecules due to their huge structures and stable state [25,26]. Thus, a solution to the peptide docking problem has the potential to foster a remarkably large number of drug development projects.

The problem of peptide docking originates from three main roots. Firstly, peptide-mediated interactions are often transient and their strength is typically weaker than that of protein–protein interactions since peptides bind to large, mostly shallow binding pockets having high structural flexibility before complexation [11,27]. Secondly, peptides have relatively large sizes and high conformational flexibility that require global search. Thirdly, peptides have many hydrophilic regions, and, therefore, they are extensively hydrated which further complicates the docking process [28,29,30,31]. Notably, the elucidation of the role of hydration and water networks is a general problem in drug design that is not restricted to peptide ligands [32,33,34,35,36,37,38,39,40]. However, incorporating explicit water molecules in molecular docking is rather challenging [41,42,43,44]. Large complexes such as adenosine A2A [45] and histone proteins [46] have been shown to be challenging due to their extensive water-mediated H-bond network with their ligands. However, only a few refinement methods are designed to incorporate experimental [47] or predicted [41] water molecules in their protocol to assist the formation of accurate mediated interactions during simulations.

Unfortunately, the above-mentioned problems of peptide docking seem to persist even in the cases of the latest methodologies based on artificial intelligence. The recent release of DeepMind’s AlphaFold2 (version 2.1.0) (AF2) [48,49] and AlphaFold-Multimer (version 2.1.0) (AFM2) [50,51] has brought the accuracy of the computational modeling of proteins to another level. Several studies have shown that both AFM2 and the input-manipulated versions of AF2 are able to predict protein–peptide complexes with high accuracy [51,52,53,54]. However, they have several major limitations including their (i) protein-only predictions, excluding cofactors, ions, or any post-translational modifications, (ii) inconsistency in the prediction quality of secondary structures and other local conformations due to their over- and under-representations during the training process [52,53], (iii) complete neglect of the effect of critical water molecules at the binding interface, (iv) decreased prediction accuracy for protein side-chains [54], and (v) inadequate modeling of conformational flexibility [49,55] which is crucial for modeling ligand binding with induced fit. A recent study also showed that despite the excellent structural agreement of their predicted ligand bound conformation to the experimental one, deep learning-based docking methods often produce physically implausible structures [56] and can be outperformed by standard, physics-based docking methods.

Thus, physics-based refinement, like molecular dynamics (MD) simulations, can help to fix the problems of structures generated by deep learning or other knowledge-based methods [57,58,59]. There have been several attempts to improve the quality of AF2 and AFM2 models using MD simulations [57,58] or applying their own recycling process when the models are used as custom template inputs [60]. The recycling route of AF2 passes the partially completed proto-model through the deep neural networks repeatedly (four times by default) [49]. Although recycling significantly improved the model quality in most cases, unrealistic atomic positions were observed [60] in the recycled models due to their unrelaxed nature. Therefore, they also suggested the combination of MD protocols and the AF2 recycling process to improve models for further applications, such as drug discovery.

Similarly, post-docking refinement steps have been also introduced in many docking protocols (Table S1). A refinement step prior to ranking could introduce structural flexibility and improve the energetics of the interface for proper scoring [61,62]. Refinements can range from short energy minimizations [63,64] removing steric clashes to more sophisticated methods that allow binding site flexibility upon ligand binding using MD [41,47,65,66,67,68,69,70,71,72] or Monte Carlo simulations [73,74]. Refinement protocols or standalone tools often include energy minimization and much longer MD simulations in nanoseconds accompanied by various optimized parameters of the simulation [41,47,68,70,71]. The docking scores are often used for ranking the refined structures accompanied by structural clustering simulation [41,47,63,64,68,70,71,73,74,75]. As MD-based protocols can effectively incorporate the effects of explicit water molecules and the flexibility of both protein target and ligand [75,76], several studies have reported the use of MD simulations for improvement of the docked poses of various ligands [41,69,70,77,78,79] including peptides [47,65,67,68,69,70,71,75,80].

While MD has proved useful in the above-mentioned structural refinements, an ultimate protocol has not been published that may be appropriate for any peptide ligands. The large variability of peptide ligands necessitates the systematic investigation of various MD parameters like simulation length, temperature, water model, restraints, clustering, etc. In this study, we focused on histone H3 peptides in complexes with their reader proteins that have crucial therapeutic, as well as diagnostic, importance in various cancers and other diseases [81,82,83,84,85,86,87,88,89,90,91,92,93]. At the same time, histones are particularly challenging ligands for molecular docking as they often interact with shallow binding pockets on the reader proteins with weak interactions measured in micromolar binding constants [94]. Moreover, their large conformational flexibility [24,95,96,97,98,99] and extensive hydration in such shallow binding pockets further complicate the prediction of accurate binding modes [24,29,99,100].

## 2. Results and Discussion

### 2.1. Systems and MD Protocols

In a previous study [94], a set of ten histone H3 peptides (Table 1) in complex with their reader proteins were used to measure the performance of eleven fast docking methods [63,64,66,73,75,94,101,102,103,104,105,106,107]. The comparison of the calculated (docked) ligand structures with the respective experimental (reference) ligand structures resulted in large root mean squared deviations (RMSD of all heavy atoms, see Section 3) of an average of 9 Å (Table S2) showing that the precision of fast docking methods is moderate for the reproduction of the histone complexes. 

**Figure 1:**
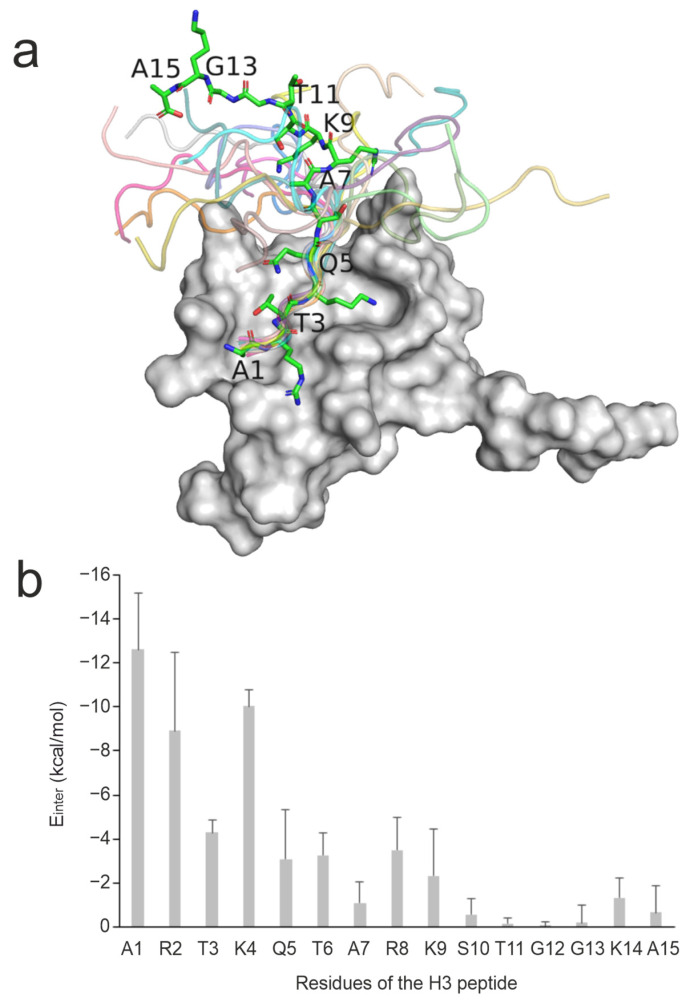
Structural and per-residue energetic analysis of histone H3 peptide bound to reader protein. (**a**) NMR solution structure of the BPTF PHD finger domain (grey surface, PDB ID 2fui) in complex with a histone H3 peptide in which the peptide structure in the first model is shown in sticks representation while the remaining models are shown in cartoon representation. (**b**) The mean (columns) and standard deviations (error bars) of E_inter_ values of all 20 NMR models calculated for the peptide respective residues upon a brief energy minimization.

The investigated complexes are particularly challenging, as mostly the N-terminal head of ca. five amino acids of the histone H3 ligand has a well-defined binding geometry, while the C-terminal region shows a high structural variability (Figure 1a). This feature can be exemplified by the high mean RMSD of 11 Å of the histone H3 ligand in the experimental solution structures of System 2fui calculated in comparison with the representative structure of the PDB entry. Thus, the nuclear magnetic resonance (NMR) spectroscopic measurements of System 2fui show the flexibility (uncertainty) of ligand conformation, especially at its C-terminal region with a high mobility in the bulk. This uncertainty can be attributed to the relatively weak interactions between the C-terminal region of histone H3 (ligand, Figure 1b) and the BPTF PHD finger (target, Figure 1b) and the correspondingly moderate (micromolar) binding affinity value (Table 1). For the same System 2fui, a moderate mean RMSD of 1 Å can be calculated for the tightly bound N-terminal region of the histone H3 ligand (Figure 1a). Accordingly, eleven fast docking methods in the previous study [94] also showed (Table S2) a better performance of an average RMSD of 7 Å calculated for the first five N-terminal amino acids if compared with the RMSD (the above-mentioned 9 Å) calculated for the entire ligand including the C-terminus.

The above-mentioned results [94] and other studies [41,45,47,56,57,58,59] concluded that complex structures of peptide ligands produced by fast docking methods have moderate structural precision, and, therefore, they should be subjected to post-docking refinements. Moreover, the ranking performance of such methods (that is the score-based selection of the close to real docked ligand structure) is very low [94].

**Figure 2:**
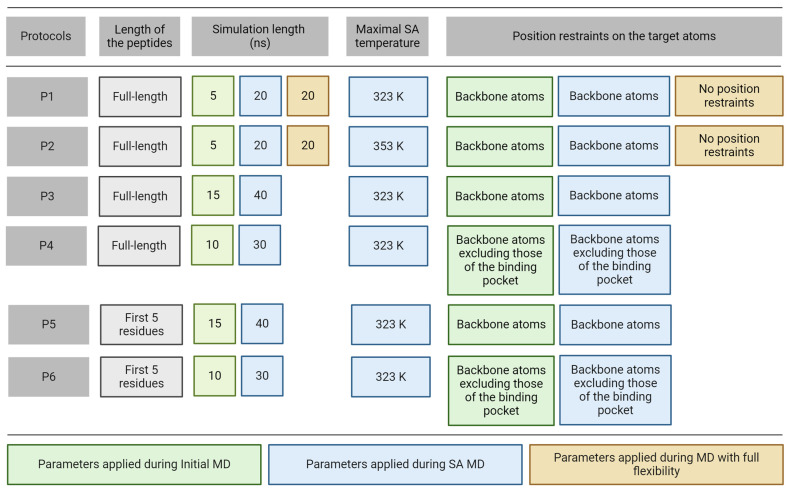
Parameters of the MD refinement protocols.

Thus, in this present study, complexes including the top-ranked docked ligand [94] conformations produced by PepGrow were used as starting structures for MD refinements. PepGrow is a protocol based on fragment-docking and constructs atomic-resolution structures of target–peptide complexes without prior knowledge of the binding site residues on the target (see also Supplementary Methods for details). Rather than attempting to link all fragments of the ligand directly, the method relies on the in situ growing of a fragment seed from the peptide ligand within the binding pocket of the reader protein. However, due to poor ranking performance, these docked ligand conformations had a relatively large RMSD (RMSD_start_, Table 1) in all complexes. Six different MD protocols were constructed from three consecutive steps (Figure 2) including initial, simulated annealing (SA), and full flexibility MD.

**Figure 3:**
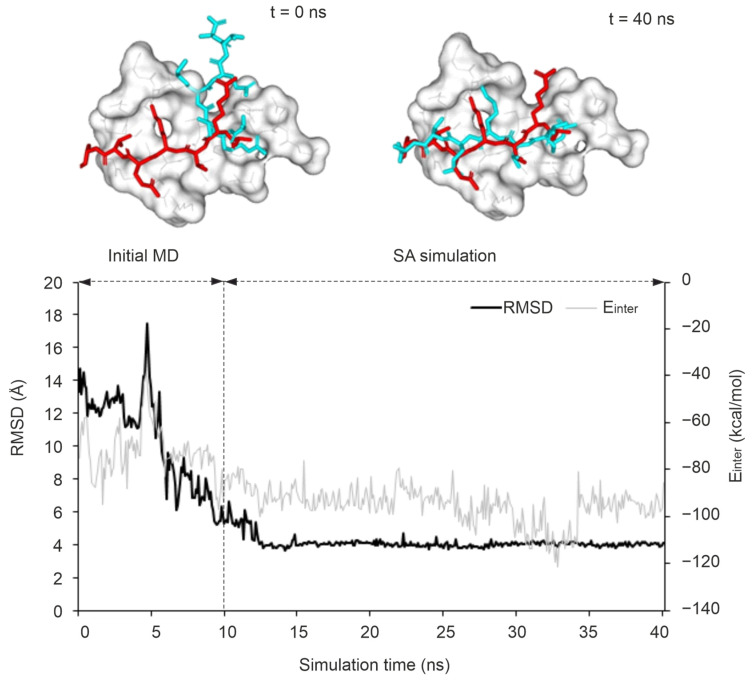
A successful MD refinement of the docked binding mode of ATRX ADD domain–histone H3 peptide complex (System 3qln) using Protocol P4. The upper plot shows the trend of RMSD of the peptide ligand and the ATRX ADD domain–peptide interaction energy (E_inter_). The best match with the experimental reference binding mode of the ligand (RMSD_best_ = 3.68 Å) was achieved after 14 ns and stabilized in the rest of the protocol. The different steps of the MD protocol are divided by a dashed line separating the first 10 ns initial MD followed by 30 ns of SA simulation. Representative structures of the complexes are shown at the top, where the experimental reference histone conformation is shown in red sticks and the starting (t = 0 ns, docked) peptide conformation, and after SA MD simulation (t = 40 ns) are shown in teal sticks. (See also Video S1 with more details on the dynamic changes).

In all cases, the same preparatory steps (Section 3) were performed prior to the MD runs including a pre-MD hydration (Section 3) that filled up the target–ligand interfaces with explicit water molecules to eliminate unwanted empty spaces. The refinement protocols differed in four parameters including temperature, position restraints, simulation time, and length of the peptide ligand (Figure 2). In Protocol P1, there were three consecutive MD simulations with an SA stage and it improved the binding mode of a pentapeptide from an RMSD of 6.6 Å (from fast docking) to 1.7 Å (after MD steps) in a previous study [80]. P1 started with a short MD simulation to remove bad interactions and to improve the target–ligand interactions, preparing the complex for further steps. It was followed by an SA MD simulation during which high temperature accelerates the thermal motion of solutes and water molecules allowing the ligand to overcome energetic barriers to explore more conformational space and move towards a minimum energy conformation as the temperature lowers. Full target flexibility in P1 and P2 did not result in significant improvement after the first two simulation steps, and, therefore, it was skipped in the next (P3–P6) protocols. Since the most notable improvements in peptide conformation, as indicated by decreased RMSD values (Figure 3 and Figure S1), occurred during the first two simulations, we extended their durations from 5 ns and 20 ns to 15 ns and 40 ns in protocols P3 and P5. Despite this adjustment, the last 5 ns of the equilibrium MD and the final 20 ns of simulated annealing MD did not show significant additional improvements (Figure S1b). Consequently, we reduced the simulation lengths to 10 ns and 30 ns in protocols P4 and P6, optimizing the balance between computational efficiency and structural refinement.

Instead of full target flexibility, the release of the binding pocket residues in P4 and P6 reduces the complexity of the simulation and sampling of irrelevant conformational space, requiring less computational time. Moreover, position restraints on the binding site surrounding residues prevent any major structural changes that could lead to instability of the complex structure. The length of each simulation was altered to ensure that peptides could find their correct positions within as minimal computational time as possible (Figure 2). All six protocols have the first two consecutive MD simulations with the peptide freely moving while position restraints were applied on different atom groups of the target to ensure optimal target flexibility during the simulations (Figure 2). A close inspection of the refined complexes showed that in some cases, extensive intramolecular interactions between the N- and C-termini of the peptide resulted in ball-like conformations limiting the development of intermolecular interactions between the peptide and the target. Thus, the C-terminal peptide tails with no role in target binding (Figure 1) were removed in Protocols P5 and P6 to foster intermolecular interactions with the target and interface water molecules instead of the intramolecular ones.

### 2.2. Structural Performance

#### 2.2.1. The Overall Performance of the MD Protocols

**Figure 4:**
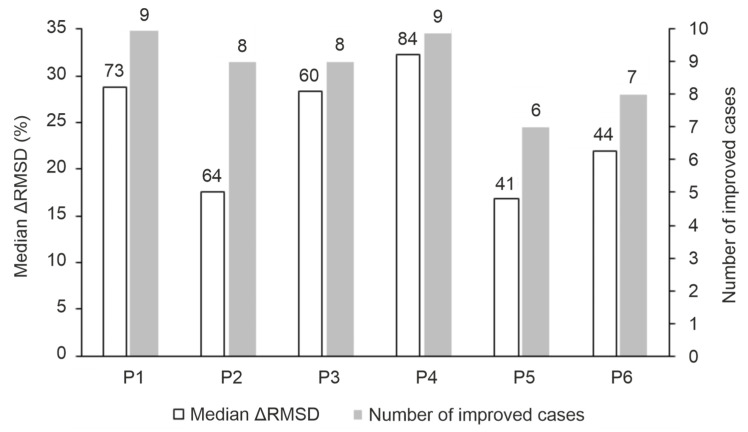
General performance of the MD refinement protocols. Empty bars indicate the median ΔRMSD (%) obtained by each protocol for the set of systems in Table 1 and the number on top shows the largest ΔRMSD (%) of the protocol. ΔRMSD (%) was calculated according to Equation (3) (Section 3). The full bars indicate the number of systems that have any improvement (ΔRMSD ≥ 0.1 Å) in a protocol and the number on top shows the number of systems with large improvements (ΔRMSD ≥ 1 Å).

The performance of the MD-based refinement protocols (Figure 2) was evaluated by measuring how close they can bring the refined ligand binding mode to the reference (experimental) one, compared to the initial fast-docked binding mode (RMSD_start_, Table S2). The improvement of starting structures (ΔRMSD), that is the decrease in RMSD upon MD refinement, is expressed in the percentage of RMSD_start_ (in Figure 4, Tables S3 and S6). The refinement protocols were able to improve the starting conformations in most cases, especially P1 and P4, displaying a large ΔRMSD of >1 Å in nearly all cases (Table S5, Figure 4). The overall statistics show that P4 outperformed all other protocols with a median ΔRMSD of 32% corresponding to a large improvement of 7.5 Å. Furthermore, P4 produced the largest improvement of 22.8 Å (84%) in the case of target TRIM24 PHD Bromo (System 3o33) starting from the largest RMSD_start_ of 27.2 Å of the test set (Table S2). Moreover, P4 was shown to improve initial conformations of relatively good quality (low RMSD_start_), as well. For example, the initial conformation of System 4qf2 obtained with its holo target structure has an RMSD_start_ of 3.8 Å. Upon P4, a large ΔRMSD improvement of 1.53 Å (40%) was observed for the system (Table S13), indicating applicability of the protocol on a wide range of starting conformations. The overall performance of Protocols P1 and P3 were comparable to that of P4, and both of them produced a median ΔRMSD close to 30% (Table S3).

The increase in simulation time in P3 (Figure 2) did not result in a significant improvement over P1 or P4 (Table S4). Notably, during longer simulations, the loosely bound (C-terminal, Figure 1) regions have more time to interact with the bulk that may result in the step-wise dissociation of the whole peptide. The increase in the maximal temperature of simulation annealing in Protocol P2 (Figure 2) was even counterproductive with a drop of median ΔRMSD below 20% except for System 4ljn. Unlike other systems (Figure 1), the C-terminal region of the histone ligand of 4ljn is engaged in extensive intramolecular interactions, resulting in a helix-like conformation. These interactions are further stabilized by weak interactions with the target. Protocols P1, P3, and P4 tend to promote stable interactions between the C-terminal region of the peptide and the target, limiting its chance to engage in intramolecular interactions. In P2, the high temperature accelerates the peptide movements minimizing its interaction with the target promoting intramolecular interactions. When the highly mobile C-terminal region of histone H3 ligands (Section 2.1) were excluded and only the N-terminal five amino acids were involved in the RMSD calculations, all protocols except P2 showed a median ΔRMSD of at least ca. 20% (Tables S6 and S7) which can be attributed to the general improvement of MD-based protocols for the strongest binding N-terminal region of the ligand (Figure 1). The above-mentioned exclusion of the non-interacting C-terminal region in Protocols P5 and P6 promoted the formation of interactions with the target and the bulk, and speeded up the optimization (drop of RMSD) for Systems 3qlc, 3o33, and 4qf2. For example, P3 was able to obtain a ΔRMSD of 22% calculated for the first five amino acids of the ligand for System 3o33. Upon removing the C-terminal region of non-specific interactions with the target, P5 achieved a ΔRMSD of 58% for the same system (Table S6).

Structural accuracy is a vital aspect of a docking method, but equally important is its ability to accurately rank binding modes. In many cases, the improvement (decrease) in RMSD was accompanied and resulted by the strengthening of the target–ligand interaction shown by the drop in the corresponding energy (E_inter_) during MD optimization (Figure 3 and Figure S1) if Protocol P4 was applied. The E_inter_ value of System 3qln shows a considerable, ca. 25% drop alongside a large ΔRMSD improvement of 9.6 Å (72%) (Figure 3, Video S1). As the terms of E_inter_ are key components of many scoring (free energy) functions [108,109], the above-mentioned improvement of E_inter_ will expectedly improve the quality of scoring and subsequent ligand ranking, which are crucial for effective drug or peptide design projects.

#### 2.2.2. The Kinetic Stability of the MD-Refined Complex Structure

The stability of an improved RMSD along the MD trajectory indicates a stable binding mode and strong interactions with a target. Several studies have reported that such kinetic stability can be used as a descriptor for discriminating real and artificial docked ligand binding modes [69,70,110]. In this present study, the kinetic stability of the correct binding mode corresponding to the largest ΔRMSD was calculated for all MD trajectories and expressed in terms of residence frequency (RF, Section 3) as it had been introduced in a previous study [111]. It was observed that systems with an RF > 0.5 ns^−1^ have a target–ligand complex of high kinetic stability along the full MD trajectory. In this sense, Protocol P4 was able to achieve the best results as 4 out of 10 systems showed high kinetic stability. As an example of a stable MD refinement, P4 improved the RMSD of the initial docked pose of System 3qln by 72% which was achieved upon a sharp drop in RMSD along the trajectory started around 5 ns into the equilibrium simulation and stabilized at 4 Å soon after entering into the final simulation (Figure 3). Compared to the trend observed in P4, the sharp drop in the RMSD started at ca. 8 ns of the SA simulation step for the same system using P1 since the target flexibility is restricted in P1, allowing an increased target motion mostly in the high temperature of SA (Figure S1a). Similarly, System 3o33 exhibited a comparable trend, with P5 (limited target flexibility) and P6 (flexible binding site region) following the same pattern (Figure S1b,c). On the other hand, for System 3qln, P2 was able to produce the best possible model with up to 41% improvement over its starting conformation after 9 ns into the SA simulation. However, the conformation did not last long due to high thermal motions (Figure S1d).

#### 2.2.3. Comparison with the Results of Other Post- and Pre-Processing Studies

Post-docking refinement methods vary in their structural performance depending on the different protocols they apply to increase the accuracy of peptide–protein docking results. A brief comparison of the performance of Protocol P4 of this present study and the best results of other methods will be discussed here. The different studies in the literature use different statistics for the measurement of the performance of the refinement. Here, we re-calculated the statistical measures of the previous studies or this present study for comparability.

The refinement protocol implemented in the HADDOCK [75,112] peptide docking protocol was reported to improve the docked ligands with a success rate of more than 15% (from 54% to 69%). The HADDOCK semi-flexible refinement works at high temperature allowing flexibility in the interface region and also performs full MD in explicit solvent. The post-docking refinement step of the peptide–protein docking method, pepATTRACT [113,114], was able to increase the initial docking success rate by 10% (from 70% to 80%) using short MD simulations with an implicit solvent model. Using the same criterion as HADDOCK and pepATTRACT, Protocol P4 of this present study improved the success rate by 20% for full-length ligands (from 10% to 30%) and for the first five amino acids (from 50% to 70%) (Section 3, Tables S8 and S9).

Accelerated MD techniques are aimed at improving conformational sampling [115,116,117]. Gaussian-accelerated MD was also used [71] to refine the global docking results of three peptide–protein complexes (with decapeptide-sized ligands) obtained by ClusPro PeptiDock [118]. Indeed, the refinement protocol produced [71] a backbone ΔRMSD of docked peptide ligands up to 83%. In the case of our Protocol P4, a maximum of 89% backbone ΔRMSD was achieved for the full H3 peptide ligand (Tables S10 and S11).

The Rosetta FlexPepDock refinement protocol was tested on 37 peptide–protein complexes (with decapeptide-sized ligands) and was able to achieve a backbone ΔRMSD of 64% for the majority of the test set [74]. The protocol uses a Monte Carlo search followed by energy minimization (EM) steps that allow full flexibility for the peptide and side-chain flexibility for the target protein. Protocol P4 (Figure 2) produced a median backbone ΔRMSD of 33% for full-length ligands (Tables S10 and S11). However, it is important to note that Rosetta FlexPepDock was tested on starting conformations with backbone RMSD_start_ of 1–5 Å while much worse geometries of RMSD_start_ values of 8–27 Å were used in this present study (Table 1) for the full-length ligand.

In addition to post-docking methods, various pre-processing approaches have been adopted in docking tools to improve prediction accuracy. Ensemble docking, for instance, incorporates target flexibility by docking ligands into either all ensemble protein structures or an average representation of these structures [119,120,121]. In peptide docking, ensemble docking has been adapted to account for peptide ligands. Specifically, tools like HPEPDOCK perform rigid-body docking of up to 1000 initial peptide conformations generated by the MODPEP program [105]. By doing so, HPEPDOCK efficiently samples the conformational flexibility of large peptide ligands while maintaining prediction accuracy. In a previous study [94], HPEPDOCK was one of the benchmark methods and showed relatively good performance with an average RMSD_best_ of 8.4 ± 3.4 Å which is comparable to that of Protocol P4 (average RMSD_best_ of 7.6 ± 2.6 Å). The main challenge in ensemble-based docking is that its success relies on the diversity and representativeness of the initial conformations. The initial set of conformations used for docking may not fully cover the true conformational space of the peptide, posing a challenge. Wang et al. reported near-native binding modes for decapeptides (with backbone RMSD of 0.6–2.7 Å) using ClusPro PeptiDock that performs global rigid body docking of an ensemble of peptide conformations retrieved from the PDB, followed by an MD refinement step [71]. Therefore, a combination of ensemble docking and MD-based post-docking refinement, like Protocol P4, can synergistically enhance the overall performance of molecular docking predictions.

### 2.3. Factors Influencing MD Refinement

#### 2.3.1. Target Conformation

The conformational state of the target may have a great impact on ligand binding and influence the accuracy of binding mode prediction [122,123,124,125]. MD provides conformational flexibility on both ligand and target sides during refinement. However, insufficient sampling may limit the performance of MD refinements. For example, the refinements failed or worked with limited accuracy in cases where the conformational change at the target interface during ligand binding exceeded an RMSD of 1–2 Å [112,114]. The apo state of the target is often characterized by a higher flexibility compared to the holo states and this higher flexibility could enable extensive conformational sampling during MD simulations [126]. In this present study, three systems (2mny, 3qln, and 4ljn) had a target conformational change at the interface above an RMSD of 2 Å (Table S12). However, the MD refinement achieved a ΔRMSD improvement of up to 72% (Protocol P4) in these three systems with starting conformations obtained using apo target structures (Tables S3 and S6). The protocols were also repeated for a docked set with holo targets that had a 2 Å better mean RMSD_start_ value (Table S13) than that of the apo set (Table 1). However, P4 produced a median ΔRMSD of 25% for the holo set while the same value was 32% for the apo set (Tables S3 and S13). These findings indicate that the MD refinement protocols are relatively robust in the sense that apo target conformations can be used, and they do not necessitate ligand-bound target conformations as starting points.

#### 2.3.2. Initial Ligand Binding Mode

**Figure 5:**
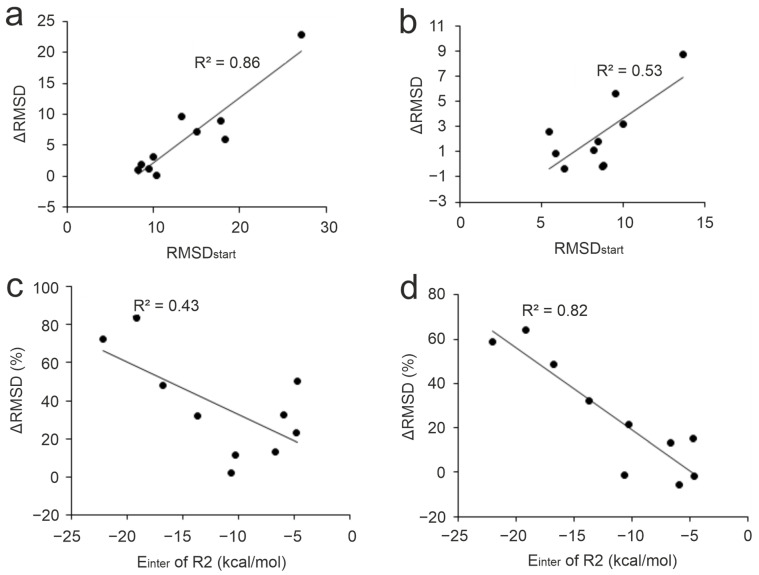
Correlations between RMSD_start_ and ΔRMSD (Protocol P4 with apo target) calculated for the full ligand (**a**), and for the N-terminal five amino acids (**b**). Correlations between E_inter_ calculated for residue R2 (initial structures) and ΔRMSD (%) calculated for full ligands (**c**) and for the N-terminal five amino acids (**d**). The data points correspond to the respective systems in Table 1.

Several refinement studies have reported the effect of the distance of the initial ligand binding mode (=position, orientation, and conformation) from the experimental (real) structure on the success of structural refinements. In the cases where the initial peptide binding mode was completely wrong, refinement procedures failed to produce the real binding mode [70,74,80]. For example, in a previous study, we carried out 1-μs-long MD simulations on a benzamidine–trypsin complex (PDB ID: 3ptb) where benzamidine was placed at three different starting positions [80]. The results showed that even in the easy case of the small benzamidine ligand, 81 ns to 690 ns of simulation time was necessary to navigate the ligand to the real binding pocket depending on the distance of the post-docking starting position from the real pocket. In this present study, moderate to high correlations (R2 of 0.86 and 0.53) were observed between RMSD_start_ and the improvement (ΔRMSD calculated for the full ligand and the N-terminal five amino acids, respectively) using Protocol P4 on the apo set (Figure 5a,b) as well as all the other protocols (R2 ranges from 0.25 to 0.73, Figure S2). Accordingly, for the N-terminal fragment, smaller improvements can be expected (see also Section 2.2). Thus, docked ligand binding modes that largely deviate from the reference (high RMSD_start_) have a high potential for improvement upon MD refinement while those close to the reference structure showed only little further improvement. This finding shows that in the case of linear peptide ligands like the N-terminal histone fragments of this present study (Table 1) MD can achieve considerable improvements even for starting situations that have a hopelessly large starting deviation from the real binding mode.

#### 2.3.3. Anchoring Residues

The presence of strong interactions between the target and the initial (docked) binding mode of the ligand affects the performance of MD refinements [68,70]. Many peptides are known to interact with their targets through highly conserved anchoring residues [27,127,128,129]. Strong interactions at these hot spots may be particularly important to drive the highly flexible, linear histone peptide to an appropriate final binding mode. In the case of histone H3 ligand, anchoring residues A1, R2, and K4 [88,90,91,92,94] are located at the N-terminal end of the peptide with the best per-residue E_inter_ (Figure 1) values. The correlations of the pre-residue E_inter_ values of these anchoring residues with ΔRMSD showed that interactions at residue R2 are the most important for a successful MD refinement, especially for the best-performing Protocol P4 (Figure 5c,d). As (the backbone of) R2 residue had an accurate initial binding mode (low RMSD_start_) in most cases (Table S14), its strong interaction with the target can keep the fragment in close proximity to its native conformation while the simulation samples conformations of the side-chain and the remaining part of the peptide. For example, the initial ligand binding mode of System 3o33 deviated largely from its experimental structure (Table 1). At the same time, anchoring R2 residue in the initial structure was relatively accurately positioned and contributed almost half of the total E_inter_ of the ligand. This provided a good starting point for sampling the possible conformations of the peptide during the simulations resulting in an 84% improvement over its RMSD_start_ with Protocol P4 (Table S3). On the other hand, System 2mny had an initial docked pose closer to its experimental structure compared to System 3o33 (Table 1). However, only weak interaction was detected between R2 residue and the target resulting in only 33% improvement upon P4 (Table S3). Thus, the correct docked (initial) binding mode of key residues like R2 is crucial for the success of post-docking MD refinements.

#### 2.3.4. Interfacial Water Network

It has been long recognized that water molecules play key roles in protein folding, stability, filling cavities, and mediating interactions with ligands [39,130]. Peptide ligands like the histone H3 fragments (Table 1) are highly hydrated, and, therefore, water molecules play a central role [32] during their binding to the target molecules. Water molecules in the interface of the binding partners can form adhesive hydrogen-bonded networks between the partners, stabilizing the protein–ligand complex structure [39,131]. However, accurately assigning all water positions in experimental structures determined by X-ray crystallography is challenging due to its limitations often rooted in the inherent mobility of water [132,133]. Other experimental methods also often suffer from improper or complete lack of water positions [133,134,135,136], necessitating the use of theoretical methods. Computational studies commonly use MD simulations with explicit solvent models to investigate the above-mentioned roles of water at the atomic level [39]. However, providing a complete hydration structure of target–ligand complexes is often challenging for the default hydration algorithms of MD simulation packages due to the restricted access of bulk water to interface regions [46] resulting in an unwanted presence of void (vacuum) cavities in the interface prior to the MD steps. In this present study, the interfaces between the docked ligand and the target molecules of all systems of Table 1 were filled with water molecules by a pre-MD hydration step based on the HydroDock protocol [41] and MobyWat [46,137] (Step 1, Section 3). This step results in a complete hydration structure without void spaces. Notably, in a previous paper [46], it was discussed that the simplicity of the default pre-MD water positioning process of MD programs and the inaccessibility of the target–ligand interface result in void spaces. That is, cavities without water molecules can remain in the interface unless such pre-MD hydration is applied as described in Step 1 of Section 3. It has also been shown [46] that the methods used in the pre-MD hydration step can produce the water structure of the interface at high precision if compared with experimental structures. For the systems of this present study (Table 1), the comparison with experimental structures was also performed. Three out of ten systems have more than one experimentally determined interfacial water molecule in the holo structures. Matches between experimental and calculated (from pre-MD hydration step) interface water positions were quantified as success rates (Section 3). The pre-MD hydration step achieved an average 86% success rate (Figure S3 and Table S15) that is in line with the previous results [46]. These findings further highlight the robustness of the hydration protocol implemented in this study.

**Figure 6:**
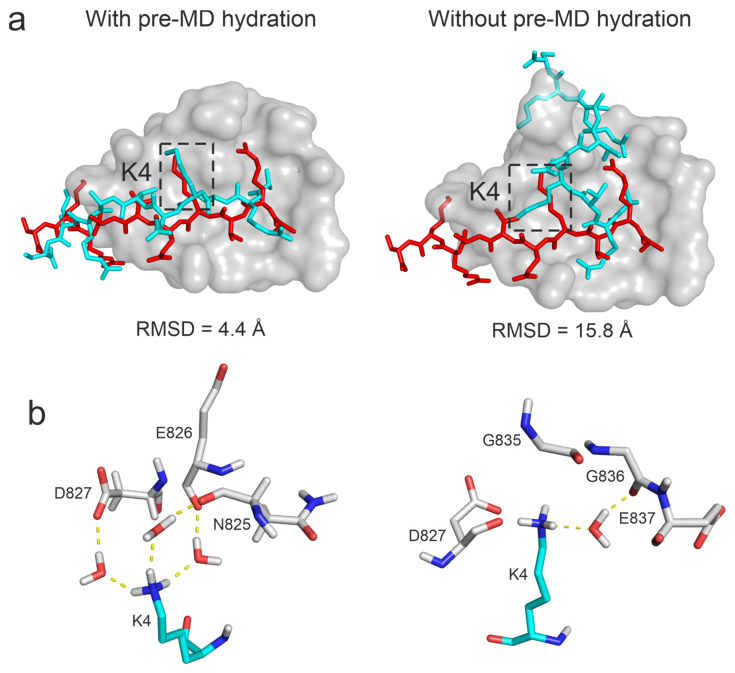
Comparison of ligand binding modes produced with and without the pre-MD hydration step (Protocol P4, System 3o33). Target, MD-refined, and experimental reference ligand structures are depicted as grey surface, cyan, and red sticks, respectively (**a**). The close-up of the surrounding of ligand residue K4 (**b**), marked by dashed boxes at the top. Three bridging water molecules can be observed if pre-MD hydration was applied, while only one water bridge was formed at a wrongly found pocket without pre-MD hydration (**b**).

To investigate the effect of the complete hydration on the results, Protocol P4 was also performed without the pre-MD hydration (Step 1, Section 3) on System 3o33, that is using only the default water positioning of the MD program. It was found that pre-MD hydration considerably improved the RMSD from 15.77 Å to 4.41 Å (that is a ΔRMSD from 42% to 84%) with Protocol P4 (Figure 6). Since the docked (starting) peptide binding mode for System 3o33 was largely deviated from the reference (Table 1), extensive conformational sampling was necessary to find its near-native conformation. However, if the pre-MD hydration was not performed, the anchoring R2 and K4 residues of the peptide formed extensive (artificial) interactions with the target limiting the movements of the peptide during the simulations (Figure 6a) and resulting in a large RMSD value. On the other hand, after pre-MD hydration, H-bond donors on the anchoring residues of the peptide were well shielded by water molecules in the starting complex structure allowing the formation of proper interactions with the target (Figure 6a) resulting in a drop of RMSD. As the pre-MD hydration fills void spaces of the interface completely, a large number of water molecules were accurately positioned with the above-mentioned shielding effect and later forming bridging hydrogen bonds between the target and the histone peptide. For example, in the case of residue K4, three bridging water molecules stabilized the interaction with the neighboring target residues in the final structure if the pre-MD hydration was applied. Without pre-MD hydration, only one water bridge was formed, providing less stability at a wrong pocket (Figure 6b). Thus, the interface water structure is crucial in promoting stable interactions and hindering the formation of artificial intramolecular interactions in the peptide. In addition to bridging hydrogen bonds, water–water interaction networks can further stabilize the target–peptide complex.

The above example showed the importance of interfacial hydration networks of large protein–peptide complexes for forming interactions between the partners, in agreement with previous studies [41,46]. This problem of correct interface hydration is vital as handling explicit water molecules is still an intractable challenge for machine learning technologies [138] and docking methods [139,140].

## 3. Methods and Materials

### 3.1. Refinement Protocols

All refinement protocols consist of two main steps: (1) pre-MD hydration (the building and equilibration of the void-free hydration structure of the complex interface) and (2) consecutive MD simulations. The uniform procedures of all steps and the common parameters of the simulations are described in the next sections, followed by the specific details of the two main steps.

Energy minimization (EM). The structure was placed in a dodecahedral box with a distance criterion of 1 nm between the solute and the box. The box was filled with explicit TIP3P water molecules [141] and counterions (sodium or chloride ions) were added to neutralize the system by the gmx solvate routine of GROMACS [142]. The simulation box was subjected to a steepest descent (sd) optimization with convergence thresholds set to 1000 kJ mol^−1^ nm^−1^. Next, conjugate gradient (cg) optimization was carried out, with convergence thresholds set to 10 kJ mol^−1^ nm^−1^. In both steps, solute-heavy atoms were position restrained at a force constant of 1000 kJ mol^−1^ nm^−2^. In cases where the target structure contained structural Zn^2+^ ions, zinc-coordinating cysteine or histidine residues had residue types with the appropriate protonation state (cysteine and histidine in the Amber force field). When position restraints were applied, the zinc ions were considered as heavy atoms of the target. All simulations were performed with the AMBER99SB-ILDN force field [143] using the GROMACS software package (version 2021.4) [142].

Molecular Dynamics (MD). After EM, NPT MD simulations were performed with a time step of 2 fs on the optimized structure. Solute and solvent were coupled separately to a reference temperature of 300 K using the velocity rescale algorithm [144] with a time constant of 0.1 ps. The temperature differs in the simulated annealing MD simulations (see below) depending on the refinement protocol (Figure 2). The pressure was kept at 1 bar using the Parrinello–Rahman algorithm [145,146,147] with a time constant of 0.5 ps and compressibility of 4.5 × 10^−5^ bar^−1^. Particle mesh Ewald summation [145] was used for long-range electrostatics using a Fourier spacing of 0.12 nm and a grid spacing of 1 Å. All van der Waals interactions were truncated at a cutoff of 11 Å. Position restraints were applied on all solute-heavy atoms with a force constant of 1000 kJ mol^−1^ nm^−2^. The bonds in solute and solvent were constrained using the LINCS [148,149] algorithm. Coordinates were saved at regular time intervals, at every 10 ps. Before analysis, periodic boundary conditions were treated and each frame was fit to the experimental target structure using Cα atoms. The final trajectory containing all atomic coordinates of all frames was saved in a portable binary file and used for subsequent procedures.

Simulated annealing (SA). During SA MD simulations, simulated annealing temperature was rescaled and controlled in the same way for each temperature group in GROMACS (both solvent and solute). For protocols (Figure 2) with a maximal SA temperature of 323 K, the simulation started at 300 K and the temperature was increased to 310 K, then to 323 K, and then cooled down to 310 K and 300 K. The simulation was performed for 2.5 ns for each temperature, except for the highest temperature. Depending on the length of the SA MD simulation of each protocol, the highest temperature was applied for the remaining simulation time (10 ns, 30 ns, 20 ns, 30 ns, and 20 ns for P1, P3, P4, P5, and P6, respectively). The maximal SA temperature was 353 K for P2 and the simulation temperature increased from 300 K to 311 K and then by 14 K intervals until it reached 353 K. From the highest temperature, it was cooled down to 300 K following the same temperature scheme. The simulation was performed for 1.2 ns for each temperature (10 ns for the highest temperature). All the other simulation parameters were unchanged and used as described in Molecular Dynamics.

Pre-MD hydration (Step 1). Hydration structures of the starting protein–peptide complexes were built using MobyWat [46,137], which predicts water positions on the target surface and in the complex interface at high precision using MD trajectories. From the starting complex structure, the ligand was removed and the resulting dry target structure was then energy minimized by sd and cg algorithms as in Energy Minimization to prepare it for the 10 ns long NPT MD simulation which was performed as described in Molecular Dynamics. Using the resulting MD trajectory file, the surface water structure of the target was calculated by the prediction mode of MobyWat using its all-inclusive identity-based prediction algorithm (IDa). During the calculation, the maximum distance from the target (dmax) of 5 Å was applied with clustering (ctol) and prediction (ptol) tolerances of 1.5 and 2.5 Å, respectively. The hydrated target structure was then placed in a common coordinate system as the starting peptide structure using Pymol [150]. The water molecules conflicting with the ligand structure were removed using the editing mode of MobyWat at a minimum distance limit (dmin) of 1.75 Å.

The hydrated complex structures were subjected to a five-step robust equilibration of the HydroDock protocol [41] to optimize the orientation of H atoms of the predicted water molecules that can assist formation of a water network. In the first two steps, sd and cg optimizations were performed as described in Energy Minimization, followed by a short 100 ps long NPT MD simulation with the same parameters as in Molecular Dynamics, with the exception that only backbone Cα atoms were position-restrained. The second round of sd and cg optimizations were carried out with the same settings as the first round, except for the position restraints which were applied on only backbone Cα atoms.

Consecutive MD simulations (Step 2). For all complex systems, three consecutive simulations of initial equilibrium MD (1), SA-MD (2), and MD with full flexibility (3) were performed on the hydrated and equilibrated complex structures depending on the refinement protocol (Figure 2). All simulations were performed with settings described in the section Molecular Dynamics, except for the parameters varied in the different protocols (Figure 2) including the simulation length, position restraints on the target atoms, SA parameters, and the length of peptide ligands. All frames generated in the MD trajectory were fitted onto the initial structure using their target Cα atoms using a GROMACS tool trjconv which also handles periodic boundary effects and centers the system in the box. Complex snapshots were extracted to individual PDB files from the resulting trajectory file by 0.1 ns steps and subjected to further analysis.

### 3.2. Evaluation Metrics

Root mean squared deviation (RMSD, Equation (1)). RMSD values were calculated between all-heavy atoms of the calculated (C in Equation (1)) and experimental reference (R) peptide binding mode according to Equation (1).

(1) $$RMSD=\sqrt{\frac{1}{NH}\sum_{i=1}^{NH}{\left|C_{i}-R_{i}\right|}^{2}}$$

NH is the number of heavy atoms in the ligand, C and R are space vectors of the ith heavy atom of the calculated and experimental reference ligand binding modes in the respective Protein Databank (PDB) coordinate files.

Improvement of RMSD. ΔRMSD values were calculated to quantify the improvement of starting structures upon MD refinement. ΔRMSD (Equation (2)) is measured as the difference between RMSD_start_ (the RMSD calculated for the starting ligand conformation obtained from docking) and RMSD_best_ (the model with the best RMSD produced by a refinement protocol, Equation (2)). The improvement is also expressed as a percentage (ΔRMSD (%) in Equation (3)) relative to RMSD_start_.

(2) $$\Delta RMSD={RMSD}_{start}-{RMSD}_{best}$$

(3) $$\Delta RMSD\left(\%\right)=\frac{\Delta RMSD\times 100}{{RMSD}_{start}}$$

The quality of IF water predictions by the pre-MD hydration step was quantified using the validation mode of MobyWat (version 1.1) [46,137] and expressed as success rates (Equation (4)).

(4) $$SR(\%)=100\frac{Count\;of\;matches}{Count\;of\;reference\;water\;positions}$$

Crystallographic positions of water molecules within a maximum distance (dmax) of 3.5 Å from both the target and ligand were used as references. A match is defined when the distance between the oxygen atoms of the predicted and reference water molecules is below the match tolerance of 1.5 Å. For detailed information on the algorithms used by MobyWat, please refer to [46,137].

Kinetic stability of a binding mode. The kinetic stability of the binding mode of a ligand during the simulation was measured by residence frequency (RF in Equation (5)) [110]. The movement of the ligand was determined by RMSD calculated between its experimental reference structure and its actual structure at each frame during the total simulation time.

(5) $$RF=\frac{Number\;of\;frames\;with\;\Delta RMSD\geq cutoff}{Simulation\;time\;(ns)}$$

The cutoff was set to 1 Å indicating that the RF value is a fraction of the simulation time in which peptide binding modes improved RMSD of the starting conformation by 1 Å or more (ΔRMSD ≥ 1 Å).

### 3.3. Per-Residue Interaction Energy Analyses

Per-residue interaction energy analysis was performed on the experimental structure of System 2fui (Figure 1). The calculations were also performed for the preparation of data in Figure 3 and Figure 5. Upon adding polar hydrogen atoms and Gasteiger–Marsilli partial charges [151] to the experimental structure, the structural file in PDB format was converted to MOL2 using OpenBabel [152]. The per-residue interaction energy (E_inter_) was then calculated on the MOL2 files according to Equation (6). The Coulomb term (E_Coulomb_) in Equation (6) was calculated with a distance-dependent dielectric function. The Lennard Jones term (E_LJ_) was calculated using Amber2012 force field parameters [153].

$$E_{inter}=E_{LJ}+E_{Coulomb}=\sum_{i,j}^{N_{T}N_{L}}\left(\frac{A_{ij}}{r_{ij}^{12}}-\frac{B_{ij}}{r_{ij}^{6}}+\frac{q_{i}q_{j}}{4\pi \varepsilon_{0}\varepsilon_{r}r_{ij}}\right)$$

$$A_{ij}=\varepsilon_{ij}R_{ij}^{12}$$

$$B_{ij}=2\varepsilon_{ij}R_{ij}^{6}$$

$$R_{ij}=R_{i}+R_{j}$$

$$\varepsilon_{ij}=\sqrt{\varepsilon_{i}\varepsilon_{j}}$$

(6) $$\varepsilon_{r}=A+\frac{B}{1+ke^{-\lambda Br}}$$

NT and NL are the number of target and ligand atoms, respectively; r_ij_ is the actual distance between the ith (ligand) and jth (target) atoms; q is the partial charge of an atom; ε_0_ is the permittivity of vacuum; ε_r_ is the distance-dependent relative permittivity; ε_ij_ is the potential well depth at equilibrium; R_ij_ is the inter-nuclear distance at equilibrium; B = ε_0_ − A in which ε_0_ is the dielectric constant of water at 25 °C; A, λ, and k are constants [154].

## 4. Conclusions

Large peptides loosely bound to their target proteins are challenging ligands for fast computational docking tools. In such cases, the application of post-docking refinement methods is necessary to achieve precise target–ligand complex structures. MD simulations have been applied for such refinements as they account for the flexibility of both target and ligand partners, and can be efficiently combined with various solvent models. In this present study, MD refinement protocols were explored using a challenging set of structures of docked complexes of histone H3 peptide ligands with a relatively large deviation from the experimental reference binding modes. In the present protocols, a pre-MD hydration step was introduced to complete the docked (starting) complex structures with a layer of water molecules. Thus, the complexes were equipped with appropriately oriented waters in the target–ligand interface and the number of unwanted void cavities was minimized prior to the MD simulations. Six different MD protocols of three consecutive MD steps and the effects of various simulation parameters were investigated. We found that MD refinements can handle the challenges of histone ligands, and the best performing Protocol P4 achieved an improvement of a median ΔRMSD of 32% (the largest improvement of 84%) if compared with the docked starting complex. Furthermore, the refinement protocols considerably improved the docked structures of large deviation from the experimental reference. An analysis of the MD parameters showed that the increase in simulation time and maximal SA temperature beyond a limit did not result in further improvement in the efficiency of the refinement. It was also concluded that an accurate positioning of anchoring residues (like R2 in the histone H3 ligand) in the docked structure considerably improves the efficiency of the MD refinement. The target–ligand intermolecular interaction energy (E_inter_) proved to be a good indicator of the quality (structural precision) of the actual complex structure during the refinements. The efficient use of (super)computational resources and the parallelized code of GROMACS have significantly reduced the time and cost associated with high-precision docking refinements to some hours using Protocol P4. Additionally, the pre-MD hydration step and the inclusion of simulated annealing within the MD protocol, and the full flexibility of the binding site region made Protocol P4 a robust option for refining initial conformations of a wide range of structural qualities. This study shows that a proper MD-based refinement protocol not only improves the structural accuracy of target–ligand complexes but also enhances the efficiency and reliability of current fast docking methods. This advancement holds a potential for accelerating the discovery of new drugs in epigenetics or any design projects working with peptide ligands.

## Figures and Tables

**Table 1:** The target–histone H3 peptide systems.

PDB ID (Apo)	Res (Å)	PDB ID (Holo)	Res (Å)	Target	Histone H3 Peptide Sequence ^1^	Kd (µM)	RMSD_start_ (Å)
1xwh	NMR ^2^	2ke1	NMR ^2^	AIRE PHD finger	ARTKQTARKS	6.5	8.56
2fui	NMR ^2^	2fuu	NMR ^2^	BPTF PHD finger	ARTKQTARKSTGGKA	2.7	17.75
2gnq	1.8	2co0	2.25	WDR5	ARTKQTARKSTGGKA	3.3	8.21
2mny	NMR ^2^	2mnz	NMR ^2^	KDM5B PHD1 finger	ARTKQTARKS	6.4	18.33
2pv0	3.3	2pvc	3.69	DNMT3L	ARTKQTA	2.1	9.51
3o33	2.0	3o37	2.0	TRIM24 PHD-Bromo complex	ARTKQTARKS	8.6	27.18
3qln	1.90	3qlc	2.5	ARTX ADD	ARTKQTARKSTGGKA	3.7	13.28
3sox	2.65	3sou	1.8	UHRF1 PHD finger	ARTKQTARK	2.1	10.32
4ljn	3.0	4lk9	1.6	MOZ double PHD finger	ARTKQTARKSTGGKAPRKQLA	-	15.02
4qf2	1.7	4q6f	1.91	BAZ2A PHD Zinc finger	ARTKQ	2.51	9.99

^1^ Experimentally determined portions of the amino acid sequences of the histone tails are underlined. ^2^ For complex structures determined by NMR, peptide structures from their first model were used as reference structures during RMSD calculations.

## Data Availability

Data files are available at https://zenodo.org/records/11051777. A compressed data file contains the in- and output files of the refinement Protocol 4 for apo systems, the scripts, and the programs necessary to produce them. A README file contains a detailed description of how to perform the protocol. The programs necessary for producing the results, MobyWat (http://mobywat.com/) and GROMACS (https://www.gromacs.org/), are open source programs. The in-house programs that were used to calculate RMSD and interaction energy values are provided in the https://zenodo.org/records/11051777.

## Supplementary Materials

The following supporting information can be downloaded at: https://www.mdpi.com/article/10.3390/ijms25115945/s1. References [150,155,156,157,158,159,160,161,162,163,164,165,166,167,168,169,170,171,172,173,174,175,176,177,178,179,180,181,182,183,184,185,186,187,188,189,190,191,192,193,194,195,196,197,198,199,200] are cited in the Supplementary Materials.
