# Supplementary material for: Efficient Refinement of Complex Structures of Flexible Histone Peptides Using Post-Docking Molecular Dynamics Protocols
Source: Int J Mol Sci. 2024 May 29;25(11):5945. doi: 10.3390/ijms25115945 (PMC11172440; doi:10.3390/ijms25115945)
Supplement: Supplementary file 1 [file ijms-25-05945-s001.zip › Supplementary_Files/ijms-3005015-supplementary.pdf]

Supplementary Material

Efficient refinement of complex structures of flexible histone peptides using post-docking molecular dynamics protocols

Bayartsetseg Bayarsaikhan, Balázs Zoltán Zsidó, Rita Börzsei, Csaba Hetényi*

Pharmacoinformatics Unit, Department of Pharmacology and Pharmacotherapy, Medical School, University of Pécs, Szigeti Út 12, 7624 Pécs, Hungary

*Corresponding author.

Supplementary Figures

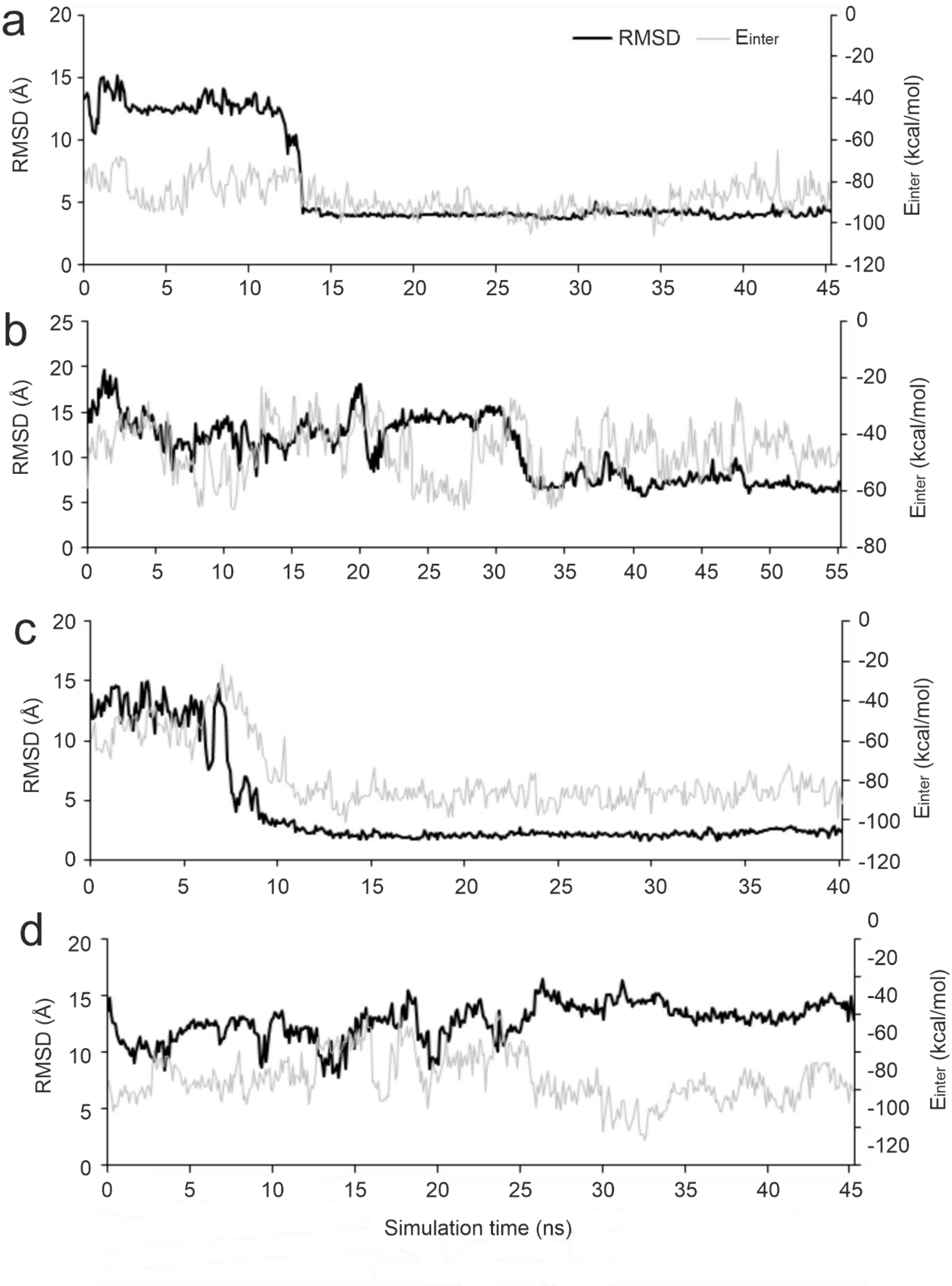

Figure S1. Examples of changes in target-peptide interaction energy (E_{inter}) and the peptide RMSD during simulations. RMSD plots changes from a starting conformation as a function of simulation time for (**a**) System 3qln produced by P1 ($RMSD_{best}$ = 3.62 Å), (**b**) System 3o33 produced by P5 ($RMSD_{best}$ = 5.75 Å), (**c**) System 3o33 produced by P6 ($RMSD_{best}$ = 1.64 Å), (**d**) System 3qln ($RMSD_{best}$ = 7.85 Å) by P2.

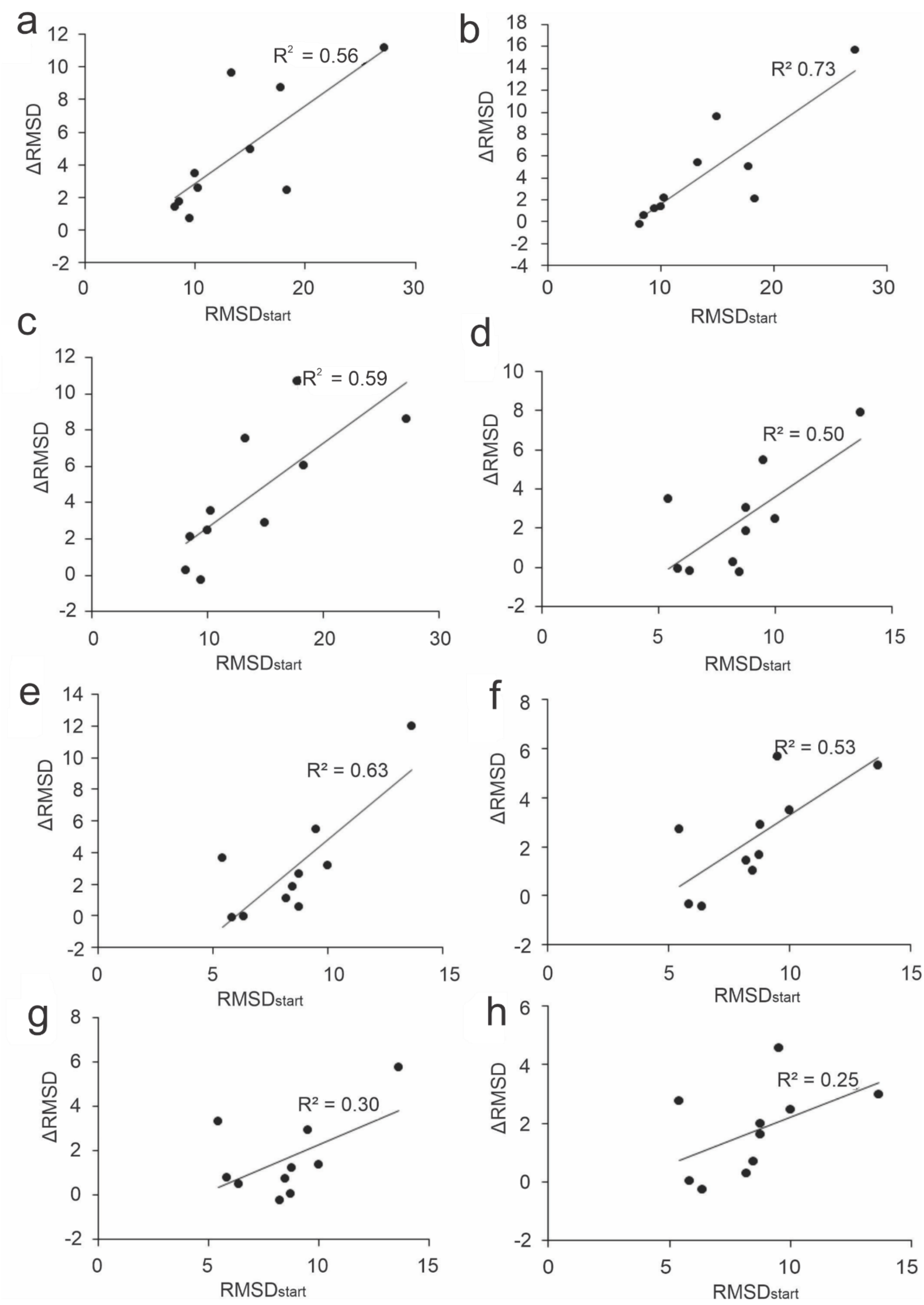

Figure S2. Correlation between $RMSD_{start}$ and ΔRMSD calculated for the full ligand obtained by (**a**) P1, (**b**) P2, (**c**) P3, (**d**) P5, (**e**) P6 and calculated for the N-terminal five amino acids obtained by (**f**) P1, (**g**) P2, (**h**) P3 using the apo set.

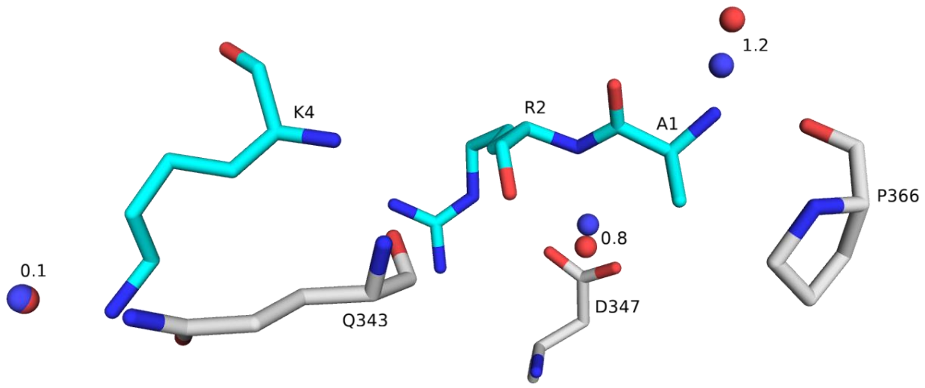

Figure S3. An example of a complete match between the experimental and predicted interface water positions, following the pre-MD hydration step, for System 3sou. Match distances between crystallographic (red spheres) and predicted (blue spheres) water oxygen atoms are given in Å. All crystallographic water positions were found at a match tolerance of 1.5 Å, resulting in a success rate of 100 % (see also Table S15). The target (UHRF1 PHD finger in holo form) and bound ligand structures are depicted as grey and cyan sticks, respectively.

Supplementary Tables

Table S1. List of refinement methods.

Author (year)	Methods	Type	Force Field	Simulation protocol	Benchmark
Standalone refinement tools/protocols					
Guterres et al. (2020) [1]	Simulation protocol	EM + MD	CGenFF [2], [3] (ligand), CHARMM 36m [4] (protein)	(i) Input file preparation with CHARMM-GUI web server [5], [6], (ii) 5000 steps sd EM followed by 1 ns NVT MD in which the ligand is positionally restrained and the protein is fully flexible, (iii) 100 ns MD runs at 303.5 K and 1 bar (2 fs time-step) with no position restraints. The MD was repeated 3 times starting with the same initial structure but different initial velocity random seeds.	Tested on 56 proteins from the DUD-E dataset [7] with five active and five decoy compounds for each protein. Focused docking was performed with AutoDock Vina [8]. AUC improved from 0.683 to 0.832 upon refinement. For 33 protein-ligand complexes with experimentally determined structures, average RMSD was improved from 5.21 Å to 4.36 Å upon refinement.
Heo et al. (2016) [9], [10]	GalaxyRefine	EM + MD	-/CHARMM 22 [11]	(i) A local EM followed by 1.3 ps-long MD with 4 fs time-step. (ii) All side-chains of the predicted binding modes were rebuilt by placing the highest-probability rotamers [12], (iii) Three MC steps were performed to repack IF residues followed by 0.6 ps-long MD with flexible side chains (4 fs time-step). Any residues within 8 Å Cα-Cα from the interacting partner considered as IF residues. The side-chain repacking and the MD simulation were repeated 22 times. The simulation temperature gradually decreased from 300 K to 50 K in the last six repetitions. The MD was performed with two different energy functions 16 times. The energy function 1 is a linear combination of CHARMM22 [11], a database-derived term and a harmonic restraint energy derived from the initial model structure. In energy function 2, the distance restraints for Cα-Cα and N-O atoms of IF residues had smaller weight constant and position restraints on all Cα were weaker than the function 1. (iv) The five lowest-energy models out of the 16 models for each energy function were selected and ranked according to their energies.	ZDOCK [13] produced 677 models for the ZDOCK benchmark 4.0 [14] set (90 hetero-complex targets) and M-ZDOCK [15] produced 445 models for the PISA benchmark set [16] (46 homo-complex targets). GalaxyRefineComplex improved the acceptable number of models by 114 for the ZDOCK benchmark set and by 75 for the PISA benchmark set.
Kapla et al. (2021) [17]	Simulation protocol*	EM + MD	ACEMD [18]/CGenFF (ligand), CHARMM 36m (protein)	(i) The ligand parameters were prepared with Schrodinger Macromodel 11.3. The receptor was placed into a POPC bilayer and solvated with TIP3P water molecules ensuring a 20 Å distance between protein periodic distances. (ii) 5000 step EM followed by 40 ns NPT MD with 2 fs time step at 1.01 bar using the Berendsen barostat with a pressure relaxation time of 800 fs and a	Tested on 30 docking results of the D3 dopamine receptor in complex with the antagonist eticlopride. Docking results submitted by 25 participants from the GPCR Dock 2010 assessment [24] were used. In terms of top-ranked models (centroids of the five largest clusters) and

				compressibility factor of 4.57x10^{-5} bar $^{-1}$. Harmonic restraints were applied on the protein backbone, the sodium ion in the transmembrane region, crystal water, and ligand heavy-atoms with 1 kcal mol^{-1} Å^{-2} in the first 20 ns and then the restraints were decreased to -0.095 kcal mol^{-1} Å^{-2} in the next 10 ns. No restraints were applied in the final 10 ns. (iii) 100 ns NVT MD (4 fs time step) was performed. The vdw interaction cut-off was set at 9 Å. For long-distance electrostatics, the Particle Mesh Ewald was used with a grid spacing of 1 Å. The bond lengths of H atoms were kept constrained using the RATTLE [19] algorithm. Simulations were carried out at 310 K using a Langevin thermostat with a damping constant γ of 1 ps^{-1} in NPT MD and 0.1 ps^{-1} in NVT MD. (iv) 1500 snapshots from trajectories of the final MD were extracted at every 200 ps. The clustering was performed with the Encore clustering module of the MDAnalysis (v.0.17.0) python package [20], [21], [22] based on ligand heavy atom RMSD by feeding an RMSD distance matrix to the affinity propagation routines from SciKit Learn [23].	RMSD of the best model, a median RMSD improvement of -0.63 Å and -1.2 Å were observed.
Lee et al. (2012) [25]	Simulation protocol	EM + MD	CHARMM/ CGenFF (ligand), CHARMM 22 [11] (protein)	(i) Equilibration MD in a 64 Å shell of TIP3P water molecules, (ii) 1000 steps sd EM followed by 1000 steps EM with the adopted Newton–Raphson method (2 fs time-step) and the SHAKE parameter was turned on. (iii) 100 ps-long NVT MD with positional restraints on protein Cα atoms and ligand heavy atoms with a harmonic force constant of 1.0 kcal mol^{-1} Å^{-2}, (iv) 300 ps-long CPT MD without any restraints was performed at 1 atm and 300 K. (iv) FEP/MD simulation was divided into 137 independent simulations, and each simulation was carried out in 10 cycles. Each cycle consisted of 10 ps equilibration and 100 ps production for repulsive, dispersive, and electrostatic contributions, 10 ps equilibration and 40 ps production for translational/rotational contributions, and 100 ps production for ligand conformational contribution.	Tested on small-molecule antagonists of MDM2 (4 compounds) and MDMX (1 compound). Focused docking was performed with AutoDock Vina [8]. AutoDock Vina SF was able to rank models with the lowest RMSD for 6 out of 10 (total docking results with apo and holo). Upon refinement, it was improved to 9 (out of 10).

Radom et al. (2018) [26]	Simulation protocol	EM + MD	NAMD [27]/CHARMM36 [28]	(i) A brief EM followed by MD with 2 fs time-step. The system was placed in a cubic box 16 Å apart from its closest image filled with TIP3P water molecules. All bonds involving H atoms were constrained. For vdw interactions, a cutoff of 12 Å and for long-range electrostatics, the particle mesh Ewald method was used. The pressure was kept constant at 1 atm using a Langevin piston, while Langevin dynamics with a low damping coefficient (1 ps^{-1}) were used to keep the temperature constant. The MD simulations started at 303 K for 32 ns and temperature increased by 30 K intervals (390 K is the highest) and the simulations performed for 12 ns for each temperature	Tested on DARPin G3 bound to domain HER2_IV and Efb-C bound to C3d. Docking was performed with RosettaDock [29] without constraints or post-processing. The top-ranked 50 docked poses were selected. For the DARPin G3 bound to HER2_IV complex, the model with the lowest RMSD was successfully ranked first upon refinement while it was ranked at 37 by the docking tool. For the Efb-C and C3d complex, the model with the lowest RMSD is ranked first successfully with the docking tool and the refinement.
Rastelli et al. (2019) [30]	Standalone tool	EM + MD	Amber/GAFF (ligand) [31], Amber ff03 [32] (protein)	(i) 2000 steps EM with a distance-dependent dielectric constant ε=4r and a cutoff of 12 Å and no restraints were applied, (ii) 100 ps MD at 300 K (2 fs time-step) with no position restraints on the ligand and the protein is fixed. The SHAKE parameter is turned on. (iii) Ranking was performed according to the binding free energies calculated with the MM-PBSA and MM-GBSA methods [33], [34], [35], [36].	Tested on 201 known inhibitors of PFDR taken from the DUD database [7] seeded in 1.5 million lead-like compounds from the ZINC database [37]. Docking was performed with AutoDock [38]. The refinement improved EF from 5.3% to 40%.
Schindler et al. (2015) [39]	Simulation protocol	EM	OPLS [40]	(i) A structure-based intramolecular protein FF was generated for each interacting partner. It consists of harmonic potentials for bond lengths, angles and a double-quadratic potential for steric repulsion between non-bonded atoms. For each atom, the steric repulsion to all neighboring atoms within a cutoff of 5 Å was considered. (ii) 2500 step-EM was performed with full interface flexibility using the structure-based FF. The interface was determined as any residues within 3 Å in the interacting partners. Each minimization step consists of small-scale rearrangements for all atoms at the interface and simultaneous large-scale translational and rotational optimization using an efficient variable metric minimizer [41], [42]. (iii) the binding modes were ranked according to their intermolecular energies.	Tested on 116 protein-protein complexes from benchmark 4.0 [14]. Rigid body docking was performed by ATTRACT [41], [42]. The average improvement of interface RMSD (all residues with backbone atoms within 10 Å from the protein partner) was -0.293 Å and a fraction of native contacts was improved by 0.071.
Wang et al. (2019) [43]	Simulation protocol	EM + GaMD	AMBER ff14SB [44]	(i) 50000 steps sd EM and 50000 steps cg EM were performed. (ii) 1 ns-long NVP MD in TIP3P water molecules where the system was heated from 0 to 310 K. 1 kcal/mol*$Å^2$ harmonic position restraints were applied on heavy atoms of the solutes. (iii) 18 ns	Tested on 3 peptide-protein complexes. Global docking was performed with ClusPro Peptidock web server protocol. For each system, models with the lowest RMSD to the experimental structure

				GaMD followed by four independent 300 ns GaMD production simulations were performed with randomized initial atomic velocities. From the final four GaMD simulations, frames were saved every 2 ps. (iv) Clustering was performed using the hierarchical agglomerative algorithm in CPPTRAJ [45] with a cutoff of 3.5 Å for the peptide backbone RMSD. The PyReweighting toolkit [46] was applied to reweight four GaMD simulations and recover PMF. The 10 top-ranked clusters of peptide conformations with the lowest free energies were obtained.	among the top-ranked 10 models were selected for refinement. Selected docked models for refinement had backbone RMSD of 3.3, 3.5, and 4.8 Å, respectively. The lowest backbone RMSDs obtained with the refinement were 0.20, 0.22, and 0.73 Å while the top-ranked models had backbone RMSDs of 0.94, 0.61, and 2.72 Å.
Refinement methods implemented in docking tools					
Kozakov et al. (2017) [47]	Implemented in ClusPro	EM	CHARMM [48]	300 step-EM with fixed backbone, using only the vdw term	-
Lamiable et al. (2016) [49]	Implemented in PEP-FOLD3	MC	-	Prior to ranking, the predicted binding modes in the sOPEP coarse-grained representation [50] are refined using 30000 MC steps.	-
Raveh et al. (2010) [51]	Implemented in PIPER FlexPepDock	MC + EM	-	10 cycles of optimization were performed and each cycle consisted of: (i) interface side-chain conformations optimization by determining the best rotamer combinations, 8 cycles of MC search performed with a Gaussian rigid body perturbation followed by Davidon-Fletcher-Powell: DFP minimization [52], (ii) optimization of the peptide backbone, 8 cycles of MC search.	Tested on 89 peptide-protein complexes (37 complexes out of 89). For starting structures with ligand RMSD ≤ 5.5 Å from the reference, near native (RMSD ≤ 2 Å) models were sampled in 91 % of the holo set and in 85% of the apo set.
Schindler et al. (2015) [53]	Implemented in pepATTRACT	EM + MD	OPLS for refinement with iATTRACT, AMBER ff14SB for refinement with AMBER	(i) iATTRACT refinement was performed with parameters specified by Schindler et al. [39] and the cutoff radius for selecting IF residues was set to 5 Å. (ii) Amber refinement, 500 step-EM by the sander program of Amber program package, (iii) two MD of 1000 and 2500 steps at 400 K and 350 K, respectively. Position restraints were applied on the intramolecular distances for the protein (2 kcal mol^{-1} $Å^{-2}$ force constant) and intermolecular distances between protein and peptide backbone atoms using a force constant of -0.25 kcal mol-1 $Å^{-2}$ for deviations more than 10 Å. (iv) 5000 step-EM without restraints. (v) Clustering was performed by the Sandar program based on the fraction of common residue contacts (cutoff = 0.6) and ranked according to the average AMBER score of their top four ranking members.	Tested on 80 peptide-protein complexes. iATTRACT refinement improved the success rate (interface RMSD < 2 Å) by 10% (average interface RMSD improvement is 0.1 Å). AMBER refinement generated one additional successful docking case compared to iATTRACT (average interface RMSD improvement is 0.44 Å).

Tovchigrechko et al. (2006) [54]	Implemented in GRAMM-X	EM	-/AMBER	Prior to ranking, a cg EM in continuous 6D rigid body space with soft vdw term.	-
Trellet et al. (2013) [55]	Implemented in HADDOCK	MD	OPLS [40]	(i) 1000 SA MD steps performed at temperatures from 2000 K to 50 K with position restraints on all heavy atoms of the solutes (8 fs time steps), (ii) SA MD performed 4000 steps from 2000 K to 50 K (4 fs time steps) with flexible side-chains at the interface [56], (iii) SA MD performed 1000 steps from 500 to 50 K (2 fs steps) with flexible interface backbone and side-chain, (iv) 500 MD steps at 100 K, 200 K, and 300 K with position restraints (kpos = 5 kcal mol^{-1} A^{-2}) on all atoms except for the interface side chains performed (2 fs time step) in an 8 Å shell filled with TIP3P [57]. (v) 5000 MD steps at 300 K with position restraints only on non-interface heavy atoms (kpos = 1 kcal mol^{-1} A^{-2}). (vi) 1000 MD steps at 300 K, 200 K, and 100 K with the position restraints on backbone atoms of non-interface residues.	Tested on 101 protein-peptide complex structures (62 complexes, apo dataset) [55]. A success rate (interface RMSD ≤ 2 Å) of rigid docking results improved from 54% to 72% upon refinement. For the apo set, the success rate was improved to 69% upon refinement.
Zsidó et al. (2021) [58]	Implemented in HydroDock*	EM + MD	GROMACS [59]/AMBER99SB-ILDN [31]	(i) The target surface water structure was predicted by MobyWat [60], [61] with clustering and prediction tolerances of 1 Å and 2.5 Å, respectively, (ii) The hydrated target structure and the docked ligand pose were merged. The clashing water molecules were removed by MobyWat, (iii) sd EM and cg EM with position restraint on solute-heavy atoms (force constant of 1000 kJ mol^{-1} nm^{-2}) were performed in a dodecahedral box filled with TIP3P (a distance criterion 1 nm). The convergence thresholds were set to 1000 and 10 kJ mol^{-1} nm^{-1} for sd and cg EM, respectively. (iv) 100 ps-long NPT MD was performed at 300 K coupled by the velocity rescale algorithm [62]. The pressure was kept at 1 bar using the Parrinello-Rahman algorithm [63], [64] and compressibility of 4.5×10^{-5} bar^{-1}. Only backbone Cα atoms were position restrained (1000 kJ mol^{-1} nm^{-2}, force constant). (v) The second round of st and cg EM were performed with the same settings as Step iii, except for the position restraints on only backbone Cα atoms. (vi) 100 ns-long NPT MD was performed with the same setting as Step iv, except that position restraints on only Cα atoms of the target. (vii) Complex snapshots were extracted from the final MD	The initial dry docking of amantadine, rimantadine and spiroadamantyl amine to the apo structure of influenza M2 transmembrane ion channel by AutoDock 4.2 [38] resulted in 3.7, 3.6 and 2.9 Å RMSD, respectively. Upon refinement, the RMSDs were improved to 1.1, 1.5 and 0.3 Å, respectively.

				trajectory by 0.1 ns steps. A binding mode with the lowest RMSD from the average ligand atomic coordinates was selected as representative binding mode.	

*Methods that incorporate water molecules

vdw, van der Waals; SF, Scoring function; FF, force field; EM, Energy minimization; MD, Molecular Dynamics; IF, interface; SA, Simulated Annealing; AUC, Area under the curve; NPT, constant pressure; NVT, conditions of constant volume; CPT, constant pressure and temperature; FEP/MD, perturbation molecular dynamics; SD, standard deviation; DARPin G3, Designed Ankyrin Repeat Protein G3; HER2_IV, IV of Human Epidermal Growth Factor Receptor 2; Efb-C, Extracellular fibrinogen-binding protein; C3d, C3-inhibitory domain of Staphylococcus aureus; GaMD, Gaussian accelerated MD; PMF, potential of mean force; EF, enrichment factor; PFDR, plasmodium falciparum dihydrofolate reductase; PME, particle mesh Ewald summation

Table S2. The performance of fast docking methods and the deviation of the docked structure (from the experimental reference) used as a starting point for MD refinements*

PDB ID	RMSD (full peptide ligand)			RMSD (first five amino acids)		
	Mean (Å)[1]	SD (Å)[1]	$RMSD_{start}$[2]	Mean (Å)[1]	SD (Å)[1]	$RMSD_{start}$[2]
1xwh	12.02	3.02	8.56	7.69	3.81	5.83
2fui	12.91	3.59	17.75	8.56	3.41	8.74
2gnq	5.18	2.42	8.21	5.18	2.42	8.21
2mny	10.97	2.13	18.33	7.58	2.08	6.36
2pv0	8.72	4.13	9.51	8.71	4.47	8.46
3o33	10.16	4.40	**27.18**	8.65	6.29	13.64
3qln	5.55	1.57	13.28	5.04	1.72	9.51
3sox	9.45	2.49	10.32	5.88	2.95	8.78
4ljn	8.97	3.61	15.02	6.84	3.99	5.42
4qf2	5.66	2.41	9.99	6.10	2.96	9.99
**Mean**	**8.96**	**2.98**	**13.82**	**7.02**	**3.41**	**8.49**
**SD**	**2.74**	**0.92**	**5.97**	**1.42**	**1.33**	**2.38**
Minimum	5.18	1.57	8.21	5.04	1.72	5.42
Maximum	12.91	4.40	27.18	8.71	6.29	13.64

*The data were extracted from [65]

[1]Mean and standard deviation (SD) of RMSD were calculated from the performance of 11 fast docking tools (AutoDock 4.2.6, CABS-DOCK, ClusPro 2.0, GalaxyPepDock, Gramm-X, HADDOCK 2.2, HDOCK, HPEPDOCK, PatchDock, PEP-FOLD3, PepGrow, and PiperFlexDock) tested in [65].

[2]$RMSD_{start}$ denotes the RMSD value calculated between the top-ranked ligand binding mode produced by PepGrow and the reference experimental structure. This top-ranked binding mode was used as a starting point in the present MD refinements.

Table S3. ΔRMSD values of the full-length peptides obtained by the protocols on the test set using apo target structure. Raw RMSD data are listed in **Table S4**.

PDB ID	ΔRMSD (Å)*						ΔRMSD (%) **					
	P1	P2	P3	P4	P5	P6	P1	P2	P3	P4	P5	P6
1xwh	1.75	0.53	2.14	1.97	-0.08	-0.14	20.48	6.19	25.00	23.01	-0.93	-1.64
2fui	8.71	5.04	10.7	8.92	1.83	0.53	49.09	28.39	60.28	50.25	10.31	2.99
2gnq	1.43	-0.26	0.28	1.10	0.28	1.10	17.39	-3.17	3.41	13.40	3.41	13.40
2mny	2.42	2.05	6.07	5.98	-0.20	-0.03	13.22	11.18	33.12	32.62	-1.09	-0.16
2pv0	0.71	1.22	-0.24	1.11	-0.26	1.85	7.46	12.83	-2.52	11.67	-2.73	19.45
3o33	11.15	15.72	8.58	**22.77**	7.89	12.00	41.02	57.84	31.57	**83.77**	29.03	**44.15**
3qln	9.66	5.44	7.54	9.60	5.49	5.50	**72.71**	40.96	**56.78**	72.29	**41.34**	41.42
3sox	2.56	2.20	3.53	0.22	3.07	2.62	24.81	21.32	34.21	2.13	29.75	25.39
4ljn	4.93	9.56	2.92	7.18	3.49	3.68	32.8	**63.65**	19.44	47.80	23.24	24.50
4qf2	3.47	1.37	2.48	3.20	2.48	3.20	34.73	13.71	24.82	32.03	24.82	32.03
Mean	4.68	4.29	4.40	6.21	2.40	3.03	31.37	25.29	28.61	36.90	15.71	20.15
SD	3.78	4.97	3.65	6.74	2.71	3.62	19.40	22.27	19.92	26.62	15.81	16.48
**Median**	**3.02**	**2.13**	**3.23**	**4.59**	**2.16**	**2.24**	**28.8**	**17.52**	**28.28**	**32.33**	**16.77**	**21.98**
MAD***	1.75	1.99	2.9	3.49	2.06	1.58	11.81	11.10	7.38	18.43	13.17	14.52

*Calculated according to Equation 2 (Methods).

** Calculated according to Equation 3 (Methods).

***Median Absolute Deviation values

Table S4. Final RMSD values of the full-length peptide obtained by the protocols on the test set using apo target structure.

PDB ID	$RMSD_{start}$	P1	P2	P3	P4	P5	P6
1xwh	8.56	6.81	8.03	6.42	6.59	5.91	5.97
2fui	17.75	9.04	12.71	7.05	8.83	6.91	8.21
2gnq	8.21	6.78	8.47	7.93	7.11	7.93	7.11
2mny	18.33	15.91	16.28	12.26	12.35	6.56	6.39
2pv0	9.51	8.80	8.29	9.75	8.40	8.72	6.61
3o33	27.18	16.03	11.46	18.60	4.41	5.75	1.64
3qln	13.28	3.62	7.85	5.74	3.68	4.02	4.01
3sox	10.32	7.76	8.12	6.79	10.10	5.71	6.16
4ljn	15.02	10.09	5.46	12.10	7.84	1.93	1.74
4qf2	9.99	6.52	8.62	7.51	6.79	7.51	6.79
Mean	13.82	9.14	9.53	9.42	7.61	6.1	5.46
SD	5.97	4.01	3.10	3.95	2.55	1.97	2.25
Median	11.80	8.28	8.38	7.72	**7.48**	6.24	6.28
MAD	3.23	1.63	0.44	1.64	1.14	0.97	0.67

Table S5 The counts of improved ligand conformations after MD refinements.

ΔRMSD (Å)	P1	P2	P3	P4	P5	P6
≥ 1.0	9	8	8	9	6	7
0.9 - 0.1	1	1	1	1	1	1
< 0.0	0	1	1	0	3	2
ΔRMSD-5 (Å)						
≥ 1.0	8	5	6	6	6	7
0.9 - 0.1	0	4	3	1	1	1
< 0.0	2	1	1	3	3	2

Table S6 ΔRMSD values of the first five amino acids of peptides obtained by the protocols on the test set using apo target structure. Raw RMSD-5 data are listed in **Table S7.**

PDB ID	ΔRMSD-5 (Å)						ΔRMSD-5 (%)					
	P1	P2	P3	P4	P5	P6	P1	P2	P3	P4	P5	P6
1xwh	-0.36	0.81	0.04	0.89	-0.08	-0.14	-6.17	13.89	0.69	15.27	-1.37	-2.40
2fui	1.68	0.05	1.61	-0.15	1.83	0.53	19.22	0.57	18.42	-1.72	20.94	6.06
2gnq	1.43	-0.26	0.28	1.10	0.28	1.10	17.42	-3.17	3.41	13.40	3.41	13.40
2mny	-0.42	0.49	-0.26	-0.34	-0.20	-0.03	-6.60	7.70	-4.09	-5.35	-3.14	-0.47
2pv0	1.02	0.75	0.68	1.84	-0.26	1.85	12.06	8.87	8.04	21.75	-3.07	21.87
3o33	5.30	5.75	2.97	8.75	7.89	12.00	38.86	42.16	21.77	64.15	57.84	87.98
3qln	5.67	2.94	4.57	5.60	5.49	5.50	59.62	30.91	48.05	58.89	57.73	57.83
3sox	2.90	1.21	2.00	-0.11	3.07	2.62	33.03	13.78	22.78	-1.25	34.97	29.84
4ljn	2.70	3.30	2.76	2.63	3.49	3.68	49.82	60.89	50.92	48.52	64.39	67.90
4qf2	3.47	1.37	2.48	3.20	2.48	3.20	34.73	13.71	24.82	32.03	24.82	32.03
Mean	2.34	1.64	1.71	2.34	2.40	3.03	25.20	18.93	19.48	24.57	25.65	31.40
SD	2.09	1.84	1.54	2.90	2.71	3.62	22.10	19.93	18.70	25.49	26.99	30.64
**Median**	**2.19**	**1.01**	**1.81**	**1.47**	**2.16**	**2.24**	**26.13**	**13.75**	**20.10**	**18.51**	**22.88**	**25.85**
MAD	1.23	0.74	1.15	1.60	2.06	1.58	13.40	9.61	14.37	19.99	25.10	23.06

Table S7. Final RMSD values of the first five amino acids of peptides obtained by the protocols on the test set using apo target structure.

PDB ID	$RMSD_{start}$	P1	P2	P3	P4	P5	P6
1xwh	5.83	6.19	5.02	5.79	4.94	5.91	5.97
2fui	8.74	7.06	8.69	7.13	8.89	6.91	8.21
2gnq	8.21	6.78	8.47	7.93	7.11	7.93	7.11
2mny	6.36	6.78	5.87	6.62	6.7	6.56	6.39
2pv0	8.46	7.44	7.71	7.78	6.63	8.72	6.61
3o33	13.64	8.34	7.89	10.67	4.89	5.75	1.64
3qln	9.51	3.84	6.57	4.94	3.92	4.02	4.01
3sox	8.78	5.88	7.57	6.78	8.89	5.71	6.16
4ljn	5.42	2.72	2.13	2.66	2.79	1.93	1.74
4qf2	9.99	6.52	8.62	7.51	6.79	7.51	6.79
Mean	8.49	6.15	6.85	6.78	6.15	6.1	5.46
SD	2.38	1.68	2.06	2.09	2.01	1.97	2.25
Median	8.6	6.65	7.64	6.96	6.66	6.24	6.28
MAD	1.15	0.61	1.02	0.9	1.75	0.98	0.67

Table S8. Final iRMSD and ΔiRMSD values of the full-length peptides obtained by the protocols on the test set using apo target structures.

PDB ID	$iRMSD_{start}$	method/minimum iRMSD values (Å)						ΔiRMSD (Å)					
		P1	P2	P3	P4	P5	P6	P1	P2	P3	P4	P5	P6
1xwh	2.35	2.54	2.51	2.32	2.30	1.48	1.47	-0.19	-0.16	0.03	0.05	-0.23	-0.22
2fui	9.15	4.68	6.29	2.84	4.41	2.12	2.91	4.47	2.86	6.31	4.74	1.09	0.29
2gnq	1.59	1.48	1.71	1.47	1.45	1.47	1.45	0.11	-0.12	0.12	0.14	0.12	0.14
2mny	6.00	5.75	5.61	4.49	3.94	1.85	1.65	0.25	0.38	1.51	2.06	-0.15	0.05
2pv0	3.42	3.26	2.95	3.42	3.39	2.15	1.37	0.16	0.47	-0.01	0.03	0.14	0.91
3o33	9.07	5.46	3.91	6.71	1.35	1.52	0.75	3.61	5.15	2.36	7.72	2.32	3.08
3qln	5.07	1.05	2.58	1.48	0.98	0.88	0.88	4.02	2.49	3.59	4.09	1.57	1.57
3sox	3.57	1.78	2.25	1.75	3.53	1.24	1.24	1.80	1.32	1.82	0.04	0.42	0.43
4ljn	6.75	4.49	2.33	5.82	2.86	0.62	0.56	2.26	4.42	0.93	3.90	0.85	0.91
4qf2	2.63	1.67	2.05	2.56	2.11	2.56	2.11	0.96	0.58	0.07	0.52	0.07	0.52
Mean	4.96	3.22	3.22	3.29	2.63	1.59	1.44	1.75	1.74	1.67	2.33	0.62	0.77
SD	2.72	1.76	1.56	1.83	1.18	0.60	0.69	1.77	1.91	2.02	2.67	0.83	0.96
Median	4.32	2.90	2.54	2.70	2.58	1.50	1.41	1.38	0.95	1.22	1.29	0.28	0.48
MAD	1.83	1.51	0.45	1.09	1.04	0.49	0.39	1.25	1.09	1.15	1.26	0.47	0.43
SR (%)*	10	40	10	30	30	70	80	-	-	-	-	-	-

*SR, success rate was determined as a percentage of systems in the test set for which a near-native binding mode (i.e. iRMSD ≤ 2 Å) was produced.

Table S9. Final iRMSD and ΔiRMSD values of the first five amino acids of peptides obtained by the protocols on the test set using apo target structure.

PDB ID	$iRMSD_{start}$	method/minimum iRMSD-5 values (Å)						ΔiRMSD-5 (Å)					
		P1	P2	P3	P4	P5	P6	P1	P2	P3	P4	P5	P6
1xwh	1.25	1.57	1.57	1.57	1.57	1.48	1.47	-0.32	-0.32	-0.32	-0.32	-0.23	-0.22
2fui	3.20	3.02	2.69	1.62	3.35	2.12	2.91	0.18	0.52	1.59	-0.15	1.09	0.29
2gnq	1.59	1.48	1.71	1.47	1.45	1.47	1.45	0.11	-0.12	0.12	0.14	0.12	0.14
2mny	1.70	1.89	1.89	1.89	1.89	1.85	1.65	-0.19	-0.19	-0.19	-0.19	-0.15	0.05

2pv0	2.29	2.00	2.07	2.29	2.07	2.15	1.37	0.29	0.21	0.01	0.21	0.14	0.91
3o33	3.84	2.54	1.95	2.91	1.26	1.52	0.75	1.30	1.89	0.93	2.58	2.32	3.08
3qln	2.45	0.91	1.43	0.94	0.91	0.88	0.88	1.55	1.02	1.51	1.54	1.57	1.57
3sox	1.66	1.25	1.61	1.42	1.65	1.24	1.24	0.42	0.06	0.24	0.01	0.42	0.43
4ljn	1.48	0.69	0.59	0.61	0.90	0.62	0.56	0.79	0.89	0.87	0.58	0.85	0.91
4qf2	2.63	1.67	2.05	2.56	2.11	2.56	2.11	0.96	0.58	0.07	0.52	0.07	0.52
Mean	2.21	1.70	1.76	1.73	1.72	1.59	1.44	0.51	0.45	0.48	0.49	0.62	0.77
SD	0.84	0.71	0.54	0.71	0.71	0.60	0.69	0.62	0.68	0.69	0.91	0.83	0.96
Median	2.00	1.62	1.80	1.60	1.61	1.50	1.41	0.35	0.37	0.18	0.18	0.28	0.48
MAD	0.49	0.38	0.24	0.48	0.41	0.49	0.39	0.49	0.51	0.44	0.36	0.47	0.43
SR (%)*	50	70	70	70	70	70	80	-	-	-	-	-	-

*SR, success rate was determined as a percentage of systems in the test set for which a near-native binding mode (i.e. iRMSD ≤ 2 Å) was produced.

Table S10. Final bbRMSD and ΔbbRMSD values of the full-length peptides obtained by the protocols on the test set using apo target structure.

PDB ID	$bbRMSD_{start}$	method/minimum bbRMSD values (Å)						ΔbbRMSD (%)					
		P1	P2	P3	P4	P5	P6	P1	P2	P3	P4	P5	P6
1xwh	6.73	6.60	6.36	5.03	6.15	4.03	4.03	1.93	5.50	25.26	8.62	-4.39	-4.39
2fui	17.89	7.48	12.18	5.36	7.41	5.21	7.17	58.24	31.92	70.04	58.58	36.97	13.09
2gnq	5.39	4.80	5.52	4.52	4.30	5.21	4.30	10.95	-2.41	16.14	20.22	16.14	20.22
2mny	17.75	15.60	15.47	11.93	11.62	4.69	4.48	12.11	12.85	32.79	34.54	1.88	6.28
2pv0	8.55	8.19	7.52	9.13	8.05	7.00	4.19	4.33	12.16	-6.78	5.85	0.99	40.74
3o33	26.83	15.60	10.43	17.92	2.82	4.35	1.12	41.86	61.16	33.21	89.49	64.83	90.95
3qln	12.81	2.32	6.44	3.65	1.97	1.94	2.14	81.89	49.73	71.51	84.62	75.22	72.81
3sox	8.53	4.75	5.91	4.53	8.33	3.84	3.82	44.31	30.72	46.89	2.34	36.84	37.17
4ljn	15.54	10.75	5.05	13.11	7.22	1.21	1.09	30.82	67.57	15.70	53.54	68.83	71.95
4qf2	8.30	4.92	6.33	6.84	5.71	6.84	5.71	40.72	23.73	17.59	31.08	17.59	31.08
Mean	12.83	8.10	8.12	8.20	6.36	4.43	3.81	32.72	29.29	32.23	38.89	31.49	37.99
SD	6.69	4.57	3.44	4.73	2.84	1.85	1.92	25.84	23.74	24.73	31.67	29.82	31.56
Median	10.68	7.04	6.40	6.10	6.69	4.52	4.11	35.77	27.23	29.02	32.81	27.22	34.13
MAD	4.41	2.27	1.00	2.02	1.50	0.77	0.99	23.07	18.39	13.10	24.98	25.78	24.44

Table S11. Final bbRMSD and ΔbbRMSD values of the first five amino acids of peptides obtained by the protocols on the test set using apo target structure.

PDB ID	$bbRMSD_{start}$	method/minimum bbRMSD-5 values (Å)						ΔbbRMSD-5 (%)					
		P1	P2	P3	P4	P5	P6	P1	P2	P3	P4	P5	P6
1xwh	3.87	4.61	4.33	4.37	4.61	4.03	4.03	-19.12	-12.14	-13.18	-19.12	-4.39	-4.39
2fui	8.25	5.12	6.66	3.44	8.48	5.21	7.17	37.94	19.27	58.30	-2.79	36.97	13.09
2gnq	5.39	4.80	5.52	4.52	4.30	5.21	4.30	10.95	-2.41	16.14	20.22	16.14	20.22
2mny	4.78	5.13	4.70	4.84	5.02	4.69	4.48	-7.32	1.67	-1.26	-5.23	1.88	6.28
2pv0	7.07	5.99	6.57	6.68	6.06	7.00	4.19	15.13	6.93	5.52	14.29	0.99	40.74
3o33	12.37	6.83	6.33	9.22	3.11	4.35	1.12	44.79	48.83	25.46	74.86	64.83	90.95
3qln	7.87	2.21	4.15	2.63	2.09	1.94	2.14	71.92	47.27	66.58	73.44	75.22	72.81
3sox	6.08	3.65	4.95	4.67	5.80	3.84	3.82	39.97	18.42	23.19	4.61	36.84	37.17

4ljn	3.85	1.52	1.14	1.15	2.33	1.21	1.09	60.52	70.39	70.13	39.74	68.83	71.95
4qf2	8.30	4.92	6.33	6.84	5.71	6.84	5.71	40.72	23.73	17.59	31.08	17.59	31.08
Mean	6.78	4.48	5.07	4.84	4.75	4.43	3.81	29.55	22.20	26.85	23.11	31.49	37.99
SD	2.59	1.62	1.67	2.29	1.93	1.85	1.92	29.01	26.09	28.88	32.04	29.82	31.56
Median	6.58	4.86	5.24	4.60	4.82	4.52	4.11	38.95	18.85	20.39	17.25	27.22	34.13
MAD	1.70	0.70	1.09	1.56	1.12	0.69	0.99	22.69	19.22	18.26	21.26	25.78	24.44

Table S12. Superimposition of experimentally determined holo and apo target structures at the interface.

PDB ID	RMSD (Å)	RMSD-Cα (Å)	RMSD** (Å)	RMSD-Cα** (Å)
1xwh*	1.65	0.97	1.82	0.84
2fui*	1.74	0.74	0.77	0.47
2gnq	0.74	0.36	1.06	0.81
2mny*	3.37	2.30	3.11	2.13
2pv0	0.93	0.61	0.95	0.73
3o33	1.17	0.69	1.58	0.61
3qln	1.25	0.80	2.74	2.88
3sox	1.26	0.64	0.89	0.72
4ljn	2.00	1.34	2.13	2.00
4qf2	0.71	0.39	1.91	1.32
Mean	1.48	0.89	1.70	1.25
SD	0.79	0.57	0.81	0.81
Median	1.26	0.72	1.70	0.83
MAD	0.44	0.18	0.70	0.29

RMSD, RMSD calculated for all heavy atoms of the targets; RMSD-Cα, RMSD calculated for Cα atoms of the targets
*For NMR structures, the first model was used for the calculations.
**RMSD calculations were performed on the binding site region, any target residues within 5 Å of the peptide ligand.

Table S13. Improvements on the full-length peptide obtained by the protocols on the test set using holo target structures.

PDB ID	$RMSD_{start}$	method/minimum RMSD values (Å)						ΔRMSD (Å)					
		P1	P2	P3	P4	P5	P6	P1	P2	P3	P4	P5	P6
1xwh	15.92	9.29	14.57	10.57	16.31	7.54	8.33	41.66	8.51	33.64	-2.43	2.46	-7.76
2fui	19.22	10.50	11.24	12.52	14.51	5.43	5.53	45.35	41.50	34.86	24.51	6.22	4.53
2gnq	7.30	6.69	6.12	5.98	5.68	5.98	5.68	8.40	16.21	18.08	22.26	18.08	22.26
2mny	10.84	6.87	7.81	7.39	8.47	7.11	7.10	36.61	27.95	31.80	21.89	-6.28	-6.14
2pv0	7.80	8.55	6.68	7.81	1.71	1.17	5.58	-9.56	14.32	-0.17	78.10	67.04	-57.04
3o33	18.28	15.12	8.36	12.78	13.74	6.18	7.40	17.29	54.29	30.10	24.82	46.12	35.48
3qln	9.13	1.98	2.30	1.39	5.19	1.16	2.12	78.32	74.80	84.75	43.11	72.71	50.16
3sox	15.06	12.83	7.99	11.41	8.75	6.68	4.48	14.83	46.97	24.25	41.92	6.31	37.18
4ljn	11.61	8.93	8.67	8.11	9.29	6.34	5.49	23.11	25.35	30.19	20.00	11.08	23.02
4qf2	3.82	1.37	3.76	2.72	2.29	2.72	2.29	64.24	1.68	28.80	40.00	28.80	40.00
Mean	11.90	8.21	7.75	8.07	8.59	5.03	5.40	32.03	31.16	31.63	31.42	25.25	14.17
SD	5.08	4.29	3.49	3.91	5.04	2.42	2.02	26.57	22.92	21.34	21.19	27.76	31.88

Median	11.23	**8.74**	**7.90**	**7.96**	**8.61**	**6.08**	**5.55**	**29.86**	**26.65**	**30.14**	**24.67**	**14.58**	**22.64**
MAD	3.88	1.96	1.50	3.03	4.28	0.84	1.31	15.26	16.49	4.11	10.00	13.17	17.74
≥ 1.0		8	9	9	9	5	6	8	9	9	9	5	6
0.9 - 0.1 Å		1	1	0	0	4	1	1	1	0	0	4	1
< 0.0 Å		1	0	1	1	1	3	1	0	1	1	1	3

Table S14. RMSD and bbRMSD calculated for residue R2 of initial structures obtained with apo targets.

PDB ID	RMSD (Å)	bbRMSD (Å)
1xwh	6.49	3.10
2fui	11.24	11.76
2gnq	8.62	2.72
2mny	7.31	2.61
2pv0	6.44	3.66
3o33	5.91	5.27
3qln	6.82	2.08
3sox	5.60	3.19
4ljn	1.88	1.56
4qf2	6.16	4.17
Mean	6.65	4.01
SD	2.36	2.92
Median	6.47	3.15
MAD	0.70	0.78

Table S15. Success Rates (SR) of the pre-MD hydration procedure.

PDB ID	Number of experimental reference waters	SR (%)
2co0	7	85.71
3sou	3	100.00
4lk9	7	71.43
Mean	5.7	85.7
SD	1.9	11.7

Supplementary Methods

Brief description of the use of PepGrow

PepGrow is a fragment-docking protocol that constructs atomic-resolution structures of target-peptide complexes without prior knowledge of the binding site residues on the target [65]. The method relies on the *in situ* growing of a fragment seed from the peptide ligand within the binding pocket of the reader protein.

1 Input preparation

All non-protein parts (ligands, waters, etc.) were removed from all selected target structures before docking. If the structure was a homo-oligomer, then only one selected chain was used (the first protein chain in the PDB file). The AR fragment of the histone H3 peptide was built with the Tinker program package [66] with the protein and xyzpdb commands. The blocking group imino-methyl (-NHMe) was added at the C-terminal end in Tinker. The acquired ligand structures were then energy minimized in Open Babel [67] with Amber99 force field [31], using a steepest descent optimization, with 10^4 steps. The next step was a conjugate gradient minimization, with a maximum of 10^4 steps, and the convergence threshold was set to 10^{-7} kcal mol^{-1} Å^{-1}.

2 Fragment docking

The fragment docking was performed by AutoDock 4.2.6 [38], [68]. The previously prepared target was handled as a rigid body while for the prepared ligand fragments, all active torsions were allowed. Gasteiger-Marsilli [69] partial charges were added to the fragment and the target structure in AutoDock Tools [68]. The number of grid points was set to 60x60x60 at a 0.375 Å grid spacing, and the middle of the box was set to the center of the respective experimental full ligand conformation. The Lamarckian genetic algorithm was used for global search. Ten docking runs were performed, and the resulting fragment conformations were ranked and the rank representatives were used in the next step.

3 Fragment growing with a homology modeling tool

Having the representative docked binding modes of the fragments produced in the previous steps, the remaining part of the H3 peptide was grown using the builder routine of the homology modeling program Modeller [70]. The docked fragment seed of the H3 peptide was placed in a common coordinate system with the target input structure (used in the docking) using PyMOL [71] and served as the template structure. The experimentally determined amino acid sequence of the histone H3-target system, recorded in the ATOM records of the corresponding PDB file, was used as the query sequence. The alignment between the template structure and the query sequence was manually optimized to ensure that the sequence of the docked dipeptide seed fit correctly with the sequence of the whole ligand. For each representative binding mode, 100 models were generated using the default building settings.

4 Scoring

The E_{inter} interaction energy score (E_{inter}, Methods) calculated for the first five amino acids was the basis of the representative model selection. As a final solution, a binding mode with coordinates closest to the average coordinates of the peptide structures ranked in the top 1% according to E_{inter} was selected as the representative peptide structure (Rank 1).

References

[1] H. Guterres and W. Im, “Improving Protein-Ligand Docking Results with High-Throughput Molecular Dynamics Simulations,” *J Chem Inf Model*, vol. 60, no. 4, pp. 2189–2198, Apr. 2020, doi: 10.1021/acs.jcim.0c00057.

[2] K. Vanommeslaeghe *et al.*, “CHARMM general force field: A force field for drug-like molecules compatible with the CHARMM all-atom additive biological force fields,” *J Comput Chem*, vol. 31, no. 4, pp. 671–690, Mar. 2010, doi: 10.1002/jcc.21367.

[3] W. Yu, X. He, K. Vanommeslaeghe, and A. D. MacKerell, “Extension of the CHARMM General Force Field to sulfonyl-containing compounds and its utility in biomolecular simulations,” *J Comput Chem*, vol. 33, no. 31, pp. 2451–2468, Dec. 2012, doi: 10.1002/jcc.23067.

[4] J. Huang *et al.*, “CHARMM36m: an improved force field for folded and intrinsically disordered proteins,” *Nat Methods*, vol. 14, no. 1, pp. 71–73, Jan. 2017, doi: 10.1038/nmeth.4067.

[5] S. Jo, T. Kim, V. G. Iyer, and W. Im, “CHARMM-GUI: a web-based graphical user interface for CHARMM,” *J Comput Chem*, vol. 29, no. 11, pp. 1859–1865, Aug. 2008, doi: 10.1002/jcc.20945.

[6] J. Lee *et al.*, “CHARMM-GUI Input Generator for NAMD, GROMACS, AMBER, OpenMM, and CHARMM/OpenMM Simulations Using the CHARMM36 Additive Force Field,” *J Chem Theory Comput*, vol. 12, no. 1, pp. 405–413, Jan. 2016, doi: 10.1021/acs.jctc.5b00935.

[7] M. M. Mysinger, M. Carchia, J. J. Irwin, and B. K. Shoichet, “Directory of useful decoys, enhanced (DUD-E): better ligands and decoys for better benchmarking,” *J Med Chem*, vol. 55, no. 14, pp. 6582–6594, Jul. 2012, doi: 10.1021/jm300687e.

[8] O. Trott and A. J. Olson, “AutoDock Vina: improving the speed and accuracy of docking with a new scoring function, efficient optimization and multithreading,” *J Comput Chem*, vol. 31, no. 2, pp. 455–461, Jan. 2010, doi: 10.1002/jcc.21334.

[9] L. Heo, H. Lee, and C. Seok, “GalaxyRefineComplex: Refinement of protein-protein complex model structures driven by interface repacking,” *Sci Rep*, vol. 6, no. 1, p. 32153, Aug. 2016, doi: 10.1038/srep32153.

[10] L. Heo, H. Park, and C. Seok, “GalaxyRefine: Protein structure refinement driven by side-chain repacking,” *Nucleic Acids Res.*, vol. 41, no. Web Server issue, pp. W384-388, Jul. 2013, doi: 10.1093/nar/gkt458.

[11] A. D. Jr. MacKerell *et al.*, “All-Atom Empirical Potential for Molecular Modeling and Dynamics Studies of Proteins,” *J. Phys. Chem. B*, vol. 102, no. 18, pp. 3586–3616, Apr. 1998, doi: 10.1021/jp973084f.

[12] R. L. Dunbrack, “Rotamer libraries in the 21st century,” *Curr Opin Struct Biol*, vol. 12, no. 4, pp. 431–440, Aug. 2002, doi: 10.1016/s0959-440x(02)00344-5.

[13] R. Chen, L. Li, and Z. Weng, “ZDOCK: an initial-stage protein-docking algorithm,” *Proteins*, vol. 52, no. 1, pp. 80–87, Jul. 2003, doi: 10.1002/prot.10389.

[14] H. Hwang, T. Vreven, J. Janin, and Z. Weng, “Protein-protein docking benchmark version 4.0,” *Proteins*, vol. 78, no. 15, pp. 3111–3114, Nov. 2010, doi: 10.1002/prot.22830.

[15] B. Pierce, W. Tong, and Z. Weng, “M-ZDOCK: a grid-based approach for Cn symmetric multimer docking,” *Bioinformatics*, vol. 21, no. 8, pp. 1472–1478, Apr. 2005, doi: 10.1093/bioinformatics/bti229.

[16] E. Krissinel and K. Henrick, “Inference of macromolecular assemblies from crystalline state,” *J Mol Biol*, vol. 372, no. 3, pp. 774–797, Sep. 2007, doi: 10.1016/j.jmb.2007.05.022.

[17] J. Kapla, I. Rodríguez-Espigares, F. Ballante, J. Selent, and J. Carlsson, “Can molecular dynamics simulations improve the structural accuracy and virtual screening performance of GPCR models?,” *PLoS Comput Biol*, vol. 17, no. 5, p. e1008936, May 2021, doi: 10.1371/journal.pcbi.1008936.

[18] M. J. Harvey, G. Giupponi, and G. D. Fabritiis, “ACEMD: Accelerating Biomolecular Dynamics in the Microsecond Time Scale,” *J. Chem. Theory Comput.*, vol. 5, no. 6, pp. 1632–1639, Jun. 2009, doi: 10.1021/ct9000685.

[19] H. C. Andersen, “Rattle: A ‘velocity’ version of the shake algorithm for molecular dynamics calculations,” *Journal of Computational Physics*, vol. 52, no. 1, pp. 24–34, Oct. 1983, doi: 10.1016/0021-9991(83)90014-1.

[20] R. Gowers *et al.*, “MDAnalysis: A Python Package for the Rapid Analysis of Molecular Dynamics Simulations,” Jan. 2016, pp. 98–105. doi: 10.25080/Majora-629e541a-00e.

[21] N. Michaud-Agrawal, E. J. Denning, T. B. Woolf, and O. Beckstein, “MDAnalysis: a toolkit for the analysis of molecular dynamics simulations,” *J Comput Chem*, vol. 32, no. 10, pp. 2319–2327, Jul. 2011, doi: 10.1002/jcc.21787.

[22] M. Tiberti, E. Papaleo, T. Bengtsen, W. Boomsma, and K. Lindorff-Larsen, “ENCORE: Software for Quantitative Ensemble Comparison,” *PLoS Comput Biol*, vol. 11, no. 10, p. e1004415, Oct. 2015, doi: 10.1371/journal.pcbi.1004415.

[23] F. Pedregosa *et al.*, “Scikit-learn: Machine Learning in Python,” *Journal of Machine Learning Research*, vol. 12, Jan. 2012.

[24] I. Kufareva, M. Rueda, V. Katritch, R. C. Stevens, R. Abagyan, and GPCR Dock 2010 participants, “Status of GPCR modeling and docking as reflected by community-wide GPCR Dock 2010 assessment,” *Structure*, vol. 19, no. 8, pp. 1108–1126, Aug. 2011, doi: 10.1016/j.str.2011.05.012.

[25] H. S. Lee, S. Jo, H.-S. Lim, and W. Im, “Application of binding free energy calculations to prediction of binding modes and affinities of MDM2 and MDMX inhibitors,” *J Chem Inf Model*, vol. 52, no. 7, pp. 1821–1832, Jul. 2012, doi: 10.1021/ci3000997.

[26] F. Radom, A. Plückthun, and E. Paci, “Assessment of ab initio models of protein complexes by molecular dynamics,” *PLoS Comput Biol*, vol. 14, no. 6, p. e1006182, Jun. 2018, doi: 10.1371/journal.pcbi.1006182.

[27] J. C. Phillips *et al.*, “Scalable molecular dynamics with NAMD,” *J Comput Chem*, vol. 26, no. 16, pp. 1781–1802, Dec. 2005, doi: 10.1002/jcc.20289.

[28] R. B. Best *et al.*, “Optimization of the additive CHARMM all-atom protein force field targeting improved sampling of the backbone φ, ψ and side-chain χ(1) and χ(2) dihedral angles,” *J Chem Theory Comput*, vol. 8, no. 9, pp. 3257–3273, Sep. 2012, doi: 10.1021/ct300400x.

[29] J. J. Gray *et al.*, “Protein-protein docking with simultaneous optimization of rigid-body displacement and side-chain conformations,” *J Mol Biol*, vol. 331, no. 1, pp. 281–299, Aug. 2003, doi: 10.1016/s0022-2836(03)00670-3.

[30] G. Rastelli and L. Pinzi, “Refinement and Rescoring of Virtual Screening Results,” *Front Chem*, vol. 7, p. 498, 2019, doi: 10.3389/fchem.2019.00498.

[31] J. Wang, R. M. Wolf, J. W. Caldwell, P. A. Kollman, and D. A. Case, “Development and testing of a general amber force field,” *J Comput Chem*, vol. 25, no. 9, pp. 1157–1174, Jul. 2004, doi: 10.1002/jcc.20035.

[32] Y. Duan *et al.*, “A point-charge force field for molecular mechanics simulations of proteins based on condensed-phase quantum mechanical calculations,” *J Comput Chem*, vol. 24, no. 16, pp. 1999–2012, Dec. 2003, doi: 10.1002/jcc.10349.

[33] B. Kuhn, P. Gerber, T. Schulz-Gasch, and M. Stahl, “Validation and use of the MM-PBSA approach for drug discovery,” *J Med Chem*, vol. 48, no. 12, pp. 4040–4048, Jun. 2005, doi: 10.1021/jm049081q.

[34] P. D. Lyne, M. L. Lamb, and J. C. Saeh, “Accurate prediction of the relative potencies of members of a series of kinase inhibitors using molecular docking and MM-GBSA scoring,” *J Med Chem*, vol. 49, no. 16, pp. 4805–4808, Aug. 2006, doi: 10.1021/jm060522a.

[35] G. Rastelli, A. Del Rio, G. Degliesposti, and M. Sgobba, “Fast and accurate predictions of binding free energies using MM-PBSA and MM-GBSA,” *J Comput Chem*, vol. 31, no. 4, pp. 797–810, Mar. 2010, doi: 10.1002/jcc.21372.

[36] S. Genheden and U. Ryde, “The MM/PBSA and MM/GBSA methods to estimate ligand-binding affinities,” *Expert Opin Drug Discov*, vol. 10, no. 5, pp. 449–461, May 2015, doi: 10.1517/17460441.2015.1032936.

[37] J. J. Irwin, T. Sterling, M. M. Mysinger, E. S. Bolstad, and R. G. Coleman, “ZINC: a free tool to discover chemistry for biology,” *J Chem Inf Model*, vol. 52, no. 7, pp. 1757–1768, Jul. 2012, doi: 10.1021/ci3001277.

[38] G. M. Morris *et al.*, “Automated docking using a Lamarckian genetic algorithm and an empirical binding free energy function,” *Journal of Computational Chemistry*, vol. 19, no. 14, pp. 1639–1662, 1998, doi: 10.1002/(SICI)1096-987X(19981115)19:14<1639::AID-JCC10>3.0.CO;2-B.

[39] C. E. M. Schindler, S. J. de Vries, and M. Zacharias, “iATTRACT: simultaneous global and local interface optimization for protein-protein docking refinement,” *Proteins*, vol. 83, no. 2, pp. 248–258, Feb. 2015, doi: 10.1002/prot.24728.

[40] W. L. Jorgensen and J. Tirado-Rives, “The OPLS [optimized potentials for liquid simulations] potential functions for proteins, energy minimizations for crystals of cyclic peptides and crambin,” *J. Am. Chem. Soc.*, vol. 110, no. 6, pp. 1657–1666, Mar. 1988, doi: 10.1021/ja00214a001.

[41] A. May and M. Zacharias, “Accounting for global protein deformability during protein-protein and protein-ligand docking,” *Biochim Biophys Acta*, vol. 1754, no. 1–2, pp. 225–231, Dec. 2005, doi: 10.1016/j.bbapap.2005.07.045.

[42] A. May and M. Zacharias, “Energy minimization in low-frequency normal modes to efficiently allow for global flexibility during systematic protein-protein docking,” *Proteins*, vol. 70, no. 3, pp. 794–809, Feb. 2008, doi: 10.1002/prot.21579.

[43] J. Wang, A. Alekseenko, D. Kozakov, and Y. Miao, “Improved Modeling of Peptide-Protein Binding Through Global Docking and Accelerated Molecular Dynamics Simulations,” *Front Mol Biosci*, vol. 6, p. 112, 2019, doi: 10.3389/fmolb.2019.00112.

[44] J. A. Maier, C. Martinez, K. Kasavajhala, L. Wickstrom, K. E. Hauser, and C. Simmerling, “ff14SB: Improving the accuracy of protein side chain and backbone parameters from ff99SB,” *J Chem Theory Comput*, vol. 11, no. 8, pp. 3696–3713, Aug. 2015, doi: 10.1021/acs.jctc.5b00255.

[45] D. R. Roe and T. E. Cheatham, “PTRAJ and CPPTRAJ: Software for Processing and Analysis of Molecular Dynamics Trajectory Data,” *J Chem Theory Comput*, vol. 9, no. 7, pp. 3084–3095, Jul. 2013, doi: 10.1021/ct400341p.

[46] Y. Miao, W. Sinko, L. Pierce, D. Bucher, R. C. Walker, and J. A. McCammon, “Improved Reweighting of Accelerated Molecular Dynamics Simulations for Free Energy Calculation,” *J Chem Theory Comput*, vol. 10, no. 7, pp. 2677–2689, Jul. 2014, doi: 10.1021/ct500090q.

[47] D. Kozakov *et al.*, “The ClusPro web server for protein-protein docking,” *Nat Protoc*, vol. 12, no. 2, pp. 255–278, Feb. 2017, doi: 10.1038/nprot.2016.169.

[48] B. R. Brooks, R. E. Bruccoleri, B. D. Olafson, D. J. States, S. Swaminathan, and M. Karplus, “CHARMM: A program for macromolecular energy, minimization, and dynamics calculations,” *Journal of Computational Chemistry*, vol. 4, no. 2, pp. 187–217, 1983, doi: 10.1002/jcc.540040211.

[49] A. Lamiable, P. Thévenet, J. Rey, M. Vavrusa, P. Derreumaux, and P. Tufféry, “PEP-FOLD3: faster de novo structure prediction for linear peptides in solution and in complex,” *Nucleic Acids Res.*, vol. 44, no. W1, pp. W449-454, Jul. 2016, doi: 10.1093/nar/gkw329.

[50] J. Maupetit, P. Derreumaux, and P. Tufféry, “A fast method for large-scale de novo peptide and miniprotein structure prediction,” *J Comput Chem*, vol. 31, no. 4, pp. 726–738, Mar. 2010, doi: 10.1002/jcc.21365.

[51] B. Raveh, N. London, and O. Schueler-Furman, “Sub-angstrom modeling of complexes between flexible peptides and globular proteins,” *Proteins*, vol. 78, no. 9, pp. 2029–2040, Jul. 2010, doi: 10.1002/prot.22716.

[52] W. C. Davidon, “Variable Metric Method for Minimization,” *SIAM J. Optim.*, vol. 1, no. 1, pp. 1–17, Feb. 1991, doi: 10.1137/0801001.

[53] C. E. M. Schindler, S. J. de Vries, and M. Zacharias, “Fully Blind Peptide-Protein Docking with pepATTRACT,” *Structure*, vol. 23, no. 8, pp. 1507–1515, Aug. 2015, doi: 10.1016/j.str.2015.05.021.

[54] A. Tovchigrechko and I. A. Vakser, “GRAMM-X public web server for protein-protein docking,” *Nucleic Acids Res.*, vol. 34, no. Web Server issue, pp. W310-314, Jul. 2006, doi: 10.1093/nar/gkl206.

[55] M. Trellet, A. S. J. Melquiond, and A. M. J. J. Bonvin, “A unified conformational selection and induced fit approach to protein-peptide docking,” *PLoS One*, vol. 8, no. 3, p. e58769, 2013, doi: 10.1371/journal.pone.0058769.

[56] C. Dominguez, R. Boelens, and A. M. J. J. Bonvin, “HADDOCK: a protein-protein docking approach based on biochemical or biophysical information,” *J Am Chem Soc*, vol. 125, no. 7, pp. 1731–1737, Feb. 2003, doi: 10.1021/ja026939x.

[57] W. L. Jorgensen, J. Chandrasekhar, J. D. Madura, R. W. Impey, and M. L. Klein, “Comparison of simple potential functions for simulating liquid water,” *The Journal of Chemical Physics*, vol. 79, no. 2, pp. 926–935, Jul. 1983, doi: 10.1063/1.445869.

[58] B. Z. Zsidó, R. Börzsei, V. Szél, and C. Hetényi, “Determination of Ligand Binding Modes in Hydrated Viral Ion Channels to Foster Drug Design and Repositioning,” *J Chem Inf Model*, vol. 61, no. 8, pp. 4011–4022, Aug. 2021, doi: 10.1021/acs.jcim.1c00488.

[59] D. Van Der Spoel, E. Lindahl, B. Hess, G. Groenhof, A. E. Mark, and H. J. C. Berendsen, “GROMACS: fast, flexible, and free,” *J Comput Chem*, vol. 26, no. 16, pp. 1701–1718, Dec. 2005, doi: 10.1002/jcc.20291.

[60] N. Jeszenői, M. Bálint, I. Horváth, D. van der Spoel, and C. Hetényi, “Exploration of Interfacial Hydration Networks of Target-Ligand Complexes,” *J Chem Inf Model*, vol. 56, no. 1, pp. 148–158, Jan. 2016, doi: 10.1021/acs.jcim.5b00638.

[61] N. Jeszenői, I. Horváth, M. Bálint, D. van der Spoel, and C. Hetényi, “Mobility-based prediction of hydration structures of protein surfaces,” *Bioinformatics*, vol. 31, no. 12, pp. 1959–1965, Jun. 2015, doi: 10.1093/bioinformatics/btv093.

[62] G. Bussi, D. Donadio, and M. Parrinello, “Canonical sampling through velocity rescaling,” *J Chem Phys*, vol. 126, no. 1, p. 014101, Jan. 2007, doi: 10.1063/1.2408420.

[63] M. Parrinello and A. Rahman, “Crystal Structure and Pair Potentials: A Molecular-Dynamics Study,” *Phys. Rev. Lett.*, vol. 45, no. 14, pp. 1196–1199, Oct. 1980, doi: 10.1103/PhysRevLett.45.1196.

[64] M. Parrinello and A. Rahman, “Polymorphic transitions in single crystals: A new molecular dynamics method,” *Journal of Applied Physics*, vol. 52, no. 12, pp. 7182–7190, Dec. 1981, doi: 10.1063/1.328693.

[65] B. Z. Zsidó, B. Bayarsaikhan, R. Börzsei, and C. Hetényi, “Construction of Histone–Protein Complex Structures by Peptide Growing,” *International Journal of Molecular Sciences*, vol. 24, no. 18, Art. no. 18, Jan. 2023, doi: 10.3390/ijms241813831.

[66] J. A. Rackers *et al.*, “Tinker 8: Software Tools for Molecular Design,” p. 37, 2019.

[67] N. M. O’Boyle, M. Banck, C. A. James, C. Morley, T. Vandermeersch, and G. R. Hutchison, “Open Babel: An open chemical toolbox,” *Journal of Cheminformatics*, vol. 3, no. 1, p. 33, Oct. 2011, doi: 10.1186/1758-2946-3-33.

[68] G. M. Morris *et al.*, “AutoDock4 and AutoDockTools4: Automated docking with selective receptor flexibility,” *J Comput Chem*, vol. 30, no. 16, pp. 2785–2791, Dec. 2009, doi: 10.1002/jcc.21256.

[69] J. Gasteiger and M. Marsili, “Iterative partial equalization of orbital electronegativity—a rapid access to atomic charges,” *Tetrahedron*, vol. 36, no. 22, pp. 3219–3228, Jan. 1980, doi: 10.1016/0040-4020(80)80168-2.

[70] A. Fiser, R. K. Do, and A. Sali, “Modeling of loops in protein structures,” *Protein Sci*, vol. 9, no. 9, pp. 1753–1773, Sep. 2000, doi: 10.1110/ps.9.9.1753.

[71] W. L. DeLano, “The PyMOL Molecular Graphics System.” Schrodinger, LLC.: New York, NY, USA, 2021.
